# Cancer literacy among Jordanian colorectal cancer survivors and informal carers: Qualitative explorations

**DOI:** 10.3389/fpubh.2023.1116882

**Published:** 2023-03-20

**Authors:** Samar J. Melhem, Shereen Nabhani-Gebara, Reem Kayyali

**Affiliations:** Department of Pharmacy, Kingston University London, Kingston upon Thames, United Kingdom

**Keywords:** cancer, health literacy, experience, patient engagement, informal carers, qualitative, health promotion (HP), Arab

## Abstract

**Introduction:**

Cancer patients face a variety of challenges in understanding their diagnosis and treatment options. Making informed decisions requires health literacy. There is scant research on how colorectal cancer (CRC) survivors and their caregivers engage with healthcare systems and obtain cancer-related knowledge to maintain proper health literacy, which is crucial for enhancing their outcomes.

**Materials and methods:**

In-depth semi-structured interviews (IDIs) with CRC survivors (*n* = 15) and online focus groups (FG) with informal caregivers (ICs) were held in Amman between Jan-June 2020. In-depth interviews were conducted using semi-structured interview protocol that addressed the healthcare experience of CRC cancer survivors. FGs evaluated ICs' perspectives of e-health for cancer care support. IDIs and FGs were done in the local Jordanian Arabic dialect, which was then translated into English. Transcribed audio-recordings were thematically coded and framework analysis was used.

**Results:**

The findings are organized around a central concept of “exploring the level of literacy and its impact.” From the overarching theme, three themes and subthemes emerged, including: (1) The current state of counseling and information provision, (2) The impact of lack of information, awareness, and literacy and (3) The health system's influence on literacy.

**Conclusions:**

Poor cancer literacy hinders patients throughout their cancer journey. Empowering cancer patients is crucial for a more timely and positive patient experience. Increased cancer literacy together with the creation of health-literate organizations and systems have the potential to improve patients' treatment throughout the continuum of care.

## Introduction

Colorectal cancer (CRC) is the third most prevalent cancer in males, the second in women, and the second major cause of cancer death worldwide (1). CRC incidence and death are declining in western nations. In Asia, Eastern Europe, and South America, CRC incidence and death have risen rapidly. The Arab population is not an exception; although having lower rates of CRC than western nations, the illness has been growing in Arab nations over the last decade (2). CRC is Jordan's second most frequent occurring malignancy and the most common among men. It is the second most prevalent cancer in women, following breast cancer (3). Across the care trajectory, CRC survivors have high and persistent cancer related information and education needs. These needs are may be due to lack of high-quality educational resources and poor information distribution modalities that meet patients' informational needs and expectations throughout the cancer trajectory (4). These requirements include information on diet, medications, dietary supplements, lifestyle changes, physical activity, sexual function, self-management of symptoms and side effects of treatment, available support groups, ability to return to work, health insurance issues, financial concerns, and life and travel insurance. In addition, studies revealed that CRC patients'/survivors' need information on the disease, its cause, therapy, surgery, stoma issues, prognosis, body image, post-surgical expectations, survival, family cancer risk, and long-term effects and follow-up of a CRC diagnosis (4, 5). In addition, they outlined that CRC survivors need to be aware of the risks associated with metastasis, recurrence, recurrence therapy, prevention, and risk reduction (4, 5).

CRC carers also have special needs throughout the cancer journey. CRC carers may develop depression, anxiety, and psychological distress similar to patients due to the stress of providing care. Such health issues may significantly impact patients' and carers' quality of life and health outcomes. Thus, CRC survivors should be supported by providing them appropriate assistance and information and education in relation to the patients they care for, their prognosis and care needs (6). To meet patients' information needs, modern healthcare models promote patient decision-making and participation. Within that context, Health literacy (HL), as a concept, becomes critical (7).

HL is a thriving area of research and practice that examines people's capacities to manage the challenging requirements of health across their lifetime (8, 9). HL is a multifaceted and heterogeneous phenomenon. The term is used to describe a person's level of proficiency in the acquisition, processing, and application of knowledge essential to making informed decisions about one's health and in promoting the health of oneself and one's community. The term “health literacy responsiveness or organizational health literacy (OHL)” refers to how well services, organizations, and systems accommodate individuals with varying levels of HL by providing them with relevant and easily accessible health information and resources (8, 10). Navigating the health care system requires navigation HL (HL-NAV), which entails choosing the right physician, communicating effectively, keeping tabs on results and findings, and starting treatment as soon as feasible. This calls for an acceptable level of HL that enables patients to navigate information according to their needs (11, 12). HL-NAV is, thus, a subset of HL. The goal of HL-NAV is to manage information so that patients can easily navigate the health-care system and “find the right care at the right time in the right place” (13). Additionally, the digitalization of healthcare systems brought digital health literacy “DHL” as a new dimension of HL. “eHealth literacy” evolved into “digital health literacy” and is now used interchangeably (14, 15). In 2006, Norman and Skinner (14) defined eHealth literacy as the ability to search, receive, comprehend, and evaluate health information online to solve health issues (14). HL underpins DHL, which involves managing Internet sourced information and disease self-management using digitally available resources (14, 15). Norman and Skinner (14) in 2006 defined DHL as a meta-competency with six sub-competences: analytical skills (literacy, numeracy, media, and information literacy) and context-specific abilities (HL, computer literacy, and scientific literacy). With the introduction of DHL, HL's curative content is expanded to include prevention-oriented material (16).

Currently, HL is interpreted using a range of definitions and conceptual frameworks. The European Health Literacy Consortium's work forms the basis for the World Health Organization's (WHO) HL model (8), which consists of 12 subdivisions relating to health promotion, prevention, and treatment. In this context, HL represents an “asset” that integrates concepts of sustainability and empowerment. According to the Edwards model (17), HL is an “asset” that develops over time as a dynamic process driven by personal, emotional, and enabling variables, and active participation in patient-provider communications and decision making (9, 17). Contrarily, according to the HL “risk” perspective, low HL is associated with a number of adverse consequences; including but not limited to: increased likelihood of illness, hospitalization, emergency room visits, failure to use preventative services, inability to comprehend health information and prescription errors, and poor health outcomes in the elderly (9). Importantly, HL is modifiable, and enhancing HL is widely seen as a means of enhancing health outcomes. Considerable evidence indicates that even in industrialized nations, HL skills are inadequate (18). The results of the first European comparative study on HL in populations conducted in eight countries, found that nearly half of respondents (47.6%) had unsatisfactory HL (19). United States (US) and Canadian studies had comparable outcomes. According to the US Department of Health and Human Services (20), 90% of participants in “the Healthy People 2030 initiative” report having difficulties using easily accessible health information in a range of contexts. Nearly two-thirds of Canadian adults and 90% of seniors lack the ability to independently access, grasp, and act on health information and services as well as make sensible health decisions (21). According to the Institute of Health Equity in the United Kingdom, 42% of English people between 16 and 65 have trouble understanding and using basic health information; this rises to 61% when numeracy is necessary (22). Many health information producers lack the skills and equipment to offer content and activities that cater to the requirements of low-literate individuals (22). Patients with cancer can face a significant informational burden related to their diagnosis and treatment (9, 23). Cancer literacy poses a unique set of obstacles compared to HL for other chronic conditions as patients are required to learn a new language of medical jargon, provide their consent for procedures, and they must know where to go and when to seek timely support (23). Since early screening, diagnosis, or treatment may have an impact on survival rates, thus a variety of time-sensitive decisions must be made by both the patient and practitioners (23). Early identification of cancer greatly improves patients' chances of survival. Treatments for cancer can be complicated, requiring interdisciplinary teams, diagnostics, medicines, and the ability to monitor and control side effects. Early diagnosis may be possible with histopathological and genetic testing, although understanding the results amidst medical jargon might be challenging (8, 9, 22). Accordingly, there may be serious clinical ramifications due to the actions (or lack thereof) of patients and healthcare providers. Further, new communication technologies and the increasing complexity of health systems have made it feasible for people to get health information instantly and continually, and “e-health literacy” is posing a challenge for patients, informal caregivers, local and international health governing bodies (8). Patients with low HL are more prone to have a fatalistic attitude toward cancer and its prevention (24), and they also have greater unmet information needs (4, 11, 15). Low HL has also been linked to avoiding medical visits and uncertainty regarding screening (10, 24, 25), which may contribute to screening avoidance. In addition, those with low HL are less likely to know about cancer screening tests including mammograms, colonoscopy, and prostate-specific antigen, and they are also less likely to accurately identify the type and specifics of cancer that is being checked for (25). In fact, the complexity and sophistication of cancer therapy can be daunting, even for patients with a sufficient level of HL. Similarly, adequate HL that spans the continuum of care from diagnosis to survivorship or end-of-life decisions are required to facilitate constructive dialogue about cancer-related issues with caregivers, family, and relatives. Despite advancements in cancer prevention and improvements in cancer survival in the general population, low cancer related HL may lessen the ability for risk management with patients being less able to manage their risks, therefore having unfavorable effects along the cancer care trajectory (11, 23). HL is therefore required for patient empowerment and can minimize health services utilization and medical expenses. Patient empowerment, the process through which individuals obtain a greater understanding of and control over their own health, is an essential component of implementing HL efforts throughout their life. The WHO has identified HL as a key social determinant of health, and the promotion of HL as a key objective of the public health sector (26). Previous qualitative research that addressed cancer related HL included patients with prostate cancer, breast cancer, and hematological malignancies (9). Nonetheless, there is a scarcity of qualitative evidence about the literacy of CRC patients, particularly in the Arabic-speaking world.

In Jordan or any of its neighboring Arab nations, there are a paucity of research examining CRC survivors' and their caregivers' experiences of healthcare and cancer-related information. Therefore, doing a study on Arabic culture in the Middle East is of significant use to bridge the existing knowledge gap. In order to improve the present healthcare system, qualitative approaches would provide valuable insights to better understand CRC patients and their ICs interactions with the healthcare system in connection to the many facets of HL. This is partly because, despite the rise of digital health information, patients still need to understand and process information to use it correctly (15). The outcomes of this study may inform the creation of evidence-based e-health interventions and educational opportunities customized to varied levels of HL to improve HL in CRC patients and their ICs.

## Materials and methods

### Ethical considerations

This study is part of a larger project to develop e-health interventions for Jordanian CRC survivors' cancer supportive care and educational needs. The study was approved by Kingston University's ethical guidelines for scientific research (approval number/1416) and Jordan University Hospital's (JUH) Internal Review Board (IRB), protocol ID (10/2019/8990). CRC survivors signed consent forms and were informed of the study's purpose before the interviews. Before the online FGs, ICs consented verbally.

### Study design and setting

This qualitative study uses the phenomenological premise of producing a textual account of what participants experience and a structural description of how they experienced it in terms of circumstances, situation, and context to investigate how survivors and ICs acquire information during healthcare encounters. Individual interviews with CRC survivors were conducted to better understand their lived experiences and show the complexity of patients' interactions with healthcare systems before, during, and after treatment. We chose one-on-one interviews with cancer patients due to the sensitive and private nature of their experiences (27). Focus groups (FGs) with ICs examined caregiving challenges and experiences. The format used enabled them to exchange ideas and generated meaningful discussions (28, 29). The FGs were carried out as a follow-up to a previous study, which showed that DHL was the only independent predictor of eHealth app use and information receptivity among CRC survivors (15). In addition, previous work identified that CRC survivors' age as an independent factor in determining their use of online information (4). Thus, in this study, we examined this phenomenon qualitatively using ICs as digital mediators. The FGs were divided into two sections; in one, ICs shared their caring experiences and in the second they brainstorm about eHealth/digital interventions and digital solution requirements, the latter section will be the subject of a separate publication.

## Data collection

### Participants and recruitment

#### CRC survivors' interviews

**Figure 1:**
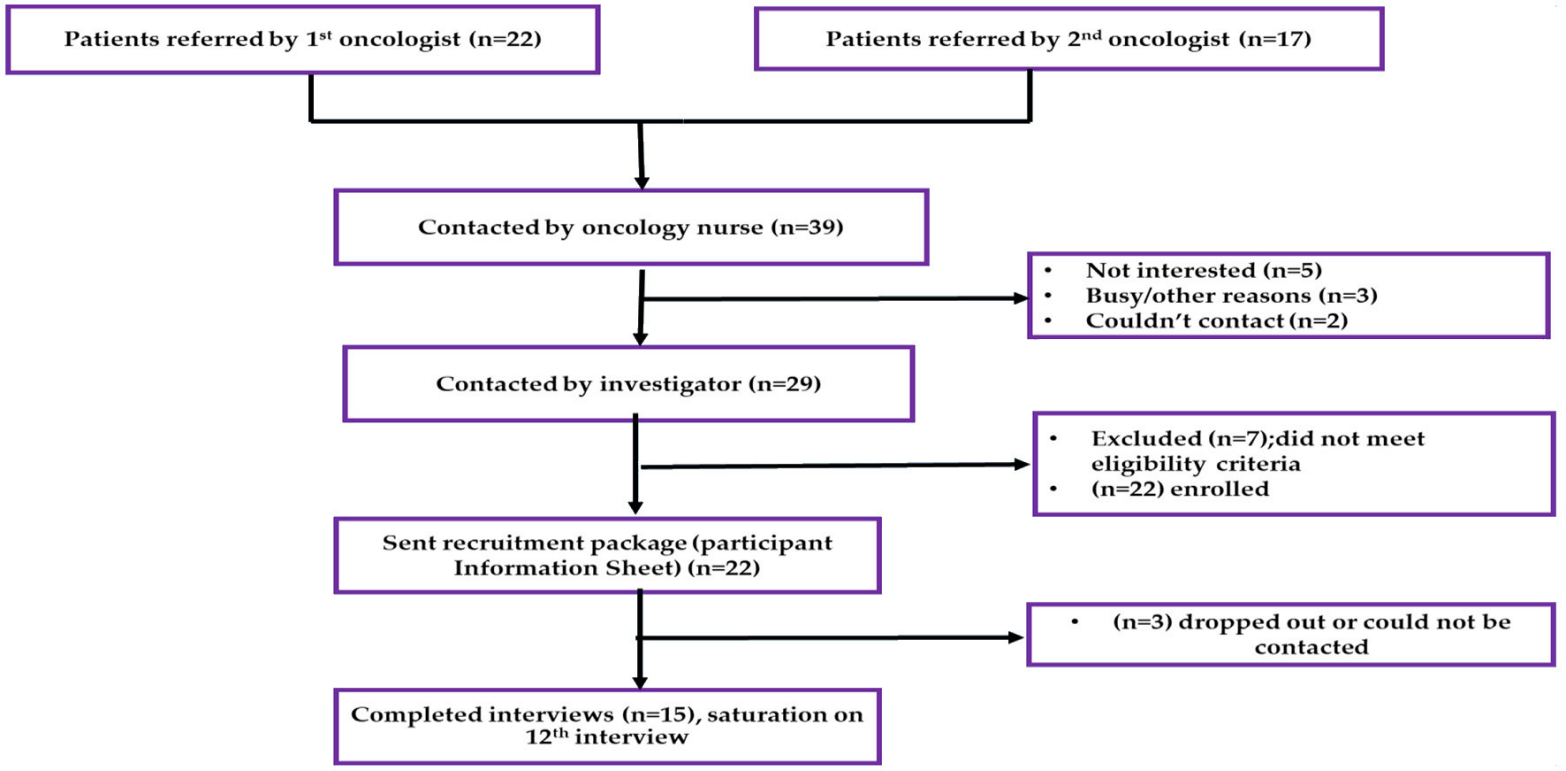
Study flow diagram.

Individual interviews were conducted with a convenient sample of ambulatory CRC survivors who had curative surgery. Demographic and clinical data were gathered from patients' electronic medical records. Participants were identified by two oncologists, and a member of the medical team contacted them. The primary investigator (SJM) contacted potential participants 1 week after the medical team to answer questions and provide study details. Interested patients received participant information sheets (PIS) *via* email or WhatsApp. Participants who met all eligibility requirements (CRC survivors eligibility shown in Table 1), were scheduled for an interview following the receipt of their consent forms *via* email or WhatsApp. Figure 1 shows the recruitment procedure.

**Table 1:** Eligibility criteria for CRC survivors' interviews and ICs' focus groups.

**CRC survivors' semi-structured interviews**	**Informal carers (ICs) focus groups**
**Eligibility criteria** ^*^	
To be an adult ≥ 18 years.	To be an adult ≥ 18 years.
Diagnosed with CRC and have finished curative therapy (i.e., are in follow-up or surveillance stage, preferably between 6 and 2 years after treatment completion).	To be a proficient Arabic speaker.
Clinically well and capable of participation as determined by the medical provider(s).	To be a current informal carer of a CRC patient.
Proficient Arabic speakers and able to give informed consent	To be digitally literate

* All criteria must be met for successful enrolment.

The first author (SJM), a qualitative female researcher (PharmD), conducted in-person interviews at JUH, a large semi-government tertiary hospital in Amman, Jordan. The semi-structured interview guide was based on an a priori framework and a literature review of CRC survivors' experiences, with an emphasis on cancer-related literacy (1–3, 30–33). An open-ended biographical framework and probing questions were used to better capture patients' complex interactions with healthcare services throughout their journey. Among the questions were: “how was your cancer diagnosed and treated?,” “tell me about the whole process and give examples” and “give me your perspectives on the whole process.” The interviews covered diagnostic routes from the patient's perspective of symptoms appraisal and help-seeking, patient-provider communications, challenges during treatment and follow-up, physical and psychosocial challenges, support and coping strategies, information acquisition, comprehension and processing, the counseling process, accessing resources and health services, and online resources. Before beginning the formal research, a pilot interview with a cancer survivor was undertaken, audiotaped and transcribed verbatim to test questions clarity, flow, and format.

The patient interview guide is included in Supplementary material 1.

### ICs' focus groups (FGs, *n* = 3)

Convenient sampling was used to recruit ICs. A member of the medical staff selected 21 ICs and contacted them to inform them of the objectives of the study. The first author (SJM) contacted the 15 carers who agreed to participate and asked if they had any additional questions before sending them the PIS and the written consent form *via* WhatsApp or email. Table 1 provides the FGs' eligibility requirements for ICs. Skype FGs with ICs were done in small groups (3–4). The FGs' guide was flexible, and probing questions were asked to delve deeper into some topics. The FGs' topic guide can be found in Supplementary material 2.

The first author (SJM) conducted all CRC survivors' interviews and ICs FGs in Jordanian Arabic, recorded them on audiotape, transcribed them verbatim in Arabic, and then translated them into English. Two Arabic-English bilingual colleagues, (RK) and (SNG), reviewed the translated transcripts for linguistic validation. (RK) and (SNG) are senior female university academics with extensive qualitative healthcare research experience. Transcripts were not returned to participants for feedback. Neither the interviews nor the FGs were witnessed by any third parties. Password-protected, de-anonymized participant data was only accessible to study team members.

## Data analysis

For the analysis, the framework methodology was applied, which included a qualitative thematic analysis with five interconnected phases for a systematic auditing process (34). Analysis consisted of familiarization, framework identification, indexing, charting, mapping, and interpretation (34, 35). Deductive-inductive hybrid analysis was used to analyze the transcripts. Inductive coding uses open (unrestricted) coding and theme refinement to derive themes from data, while deductive coding uses pre-selected themes and codes based on previous literature and preconceived ideas about the research subject (34, 35). A small sample of interview transcripts were read and reread to search for data patterns. A coding approach was developed to identify themes and correlations between qualitative data extracts, revealing a pattern in how CRC survivors and their ICs interact with healthcare systems across the cancer continuum. This was done by evaluating the different lenses and dimensions of HL and how it may affect survivors' literacy, as well as any other key considerations related to the study objectives. Due to the heterogeneity of HL's conceptualizations and definitions, the analysis used a priori concepts and definitions, but new themes were also constructed inductively where data could not be accommodated by the framework, in order to avoid forcing data into predetermined categories (35). The “HL Pathway Model and HL Skills Framework” was used as a foundation for a priori analytical framework to analyse data on HL skills and develop strategies to improve HL in cancer survivors (17, 36–38). Previous model by Edwards et al. (17) defines HL development as a lengthy process influenced by personal, emotional, and enabling factors that culminates in dialogues and collaborative decision making. HL is regarded as an “asset” for cancer patients' decision-making since they must make challenging decisions (17). Pre-diagnostic findings were analyzed using the theoretical framework of the Pathways to treatment model to reach similar conclusions to other early cancer detection studies (36, 37). This model examines the characteristics of patients, providers, the system, and illnesses in four stages: (symptom) evaluation, help-seeking, diagnostics, and pre-treatment. Even though it acknowledges that patients may consult with different healthcare providers at different times, the process is typically depicted as following a linear path that ends with a diagnosis from healthcare providers (HCPs). Inductive open (unrestricted) coding was used to address culture, participant experiences, health care system contextual components, and unexpected features of participant experiences or how they ascribe meanings to events (34). Deductive themes refined inductive codes. The analysis was done iteratively, for instance, the authors' review and revision of the existing themes resulted in the emergence of a number of new themes (34). All authors verified the final themes and subthemes to ensure data analytic bias, validity of interpretations, and consistency of findings.

## Quality appraisal and rigor

The Consolidated Criteria for Reporting Qualitative (COREQ) Research reporting list was used for methodological rigor (Supplementary material 3) (39). Recording and transcribing interviews, using a flexible interview guide to examine participants' perspectives, and holding regular research meetings to discuss results ensured data credibility and transferability (40, 41). Data integrity was ensured by transparent data collection, management, and analysis (42). Direct quotations, sample and context descriptions (43), and connections to prior research serve transferability (43–45).

## Results

### Participant characteristics

#### CRC survivors' individual interviews

Between January 15 and February 28, 2020, interviews with 15 participants were conducted. The median age was 57 and the average was 55.4 (range 33–72). The average interview lasted 72 min (range 55–112). All participants were married. Data saturation was used to determine how many interviews were required for reliable results. After no new themes emerged, three more interviews were done to ensure thematic saturation (43). Most participants (*n* = 9) were medium-term survivors, and the median time after surgery was 11 months (range: 6–22). The majority were diagnosed at stage 3. Participants' characteristics are provided in Table 2.

**Table 2:** Characteristics of participants of semi-structured interviews (*n* = 15).

**Variable(s)**	**CRC survivors (*N* = 15)**
	***N*** **(%)**
Age (years)^a^	58 (33–72)
Male(s)	9 (60)
Female (s)	6 (40)
**Education**	
Primary (5–8 years)	0 (0)
Secondary (9–12 years)	2 (13.3)
High school/collage/diploma (12+ years)	6 (40)
University (14+)	7 (46.6)
**Employment**	
Employed	6 (40)
Unemployed (capable/uncapable)	7 (46.7)
Retired	2 (13.3)
**Cancer type**	
Colon	12 (80)
Rectal	3 (20)
**Cancer stage**	
Stage I	0 (0)
Stage II	5 (33)
Stage III	8 (54)
Stage IV	2 (13)
**Treatment modality**	
Chemotherapy, surgery	9 (60)
Chemoradiation, surgery	4 (26.6)
Chemotherapy, surgery, palliative chemotherapy	1 (6.7)
Surgery	1 (6.7)
**Stoma**	
None	5 (33)
Temporary, reversed	8 (54)
Permanent	1 (6.7)
Unknown	1 (6.7)
**Route of diagnosis**	
Self-led	3 (20)
Multiple point of contact	5 (33.3)
Incidental	2 (13.3)
Emergency admission	5 (33.3)
Time since diagnosis (years)^a^	2 (1–5)
Time since surgery (months)^a^	11 (6–22)
**Comorbidities**	
Yes	6 (40)
No	9 (60)

a Median (min, max).

#### Informal carers' focus groups

Between 12/6/2020 and 28/6/2020, three FGs with a total of 10 ICs were held online [FG number (hour: minutes)]: FG1 (2:47), FG 2 (3:02), FG 3 (2:51). Table 3 shows participants' characteristics. The concept of theoretical saturation of themes was used when the third FG did not generate new ideas. Hence further research such as analyzing data from a fourth focus group session was not pursued, and the data obtained was deemed sufficient to meet the study objectives (46). According to Guest et al. (47), theoretical saturation can be guided by the assumption that conducting 2–3 FGs with a semi–structured guide in a relatively homogeneous population will likely capture at least 80% of the themes on a topic, including the most popular ones, with three to six FGs identifying 90% of themes. In this case, the third FG outlined the reach of saturation.

**Table 3:** Characteristics of participants of focus groups (*n* = 3).

**Variable(s)**	**Informal carers (*N* = 10)**
	***N*** **(%)**
Age (years)^a^	36 (26–62)
Male(s)	4 (40)
Female (s)	6 (60)
**Education**	
Primary (5–8 years)	0 (0)
Secondary (9–12 years)	0 (0)
High school/collage (12+ years)	1 (10)
University (14+years)	3 (30)
Masters/Ph.D. (18+ years)	6 (60)
**Occupation**	
Medical professional	5 (50)
Engineering, design, tourism	3 (30)
Academia	1 (10)
Housewife	1 (10)
**Patients' cancer type**	
Colon	8 (80)
Rectal	2 (20)
**Relationship of carer-patient**	
Son/daughter	7 (70)
Stepmother	1 (10)
Spouse	1 (10)
Sibling	1 (10)
**Time since patients' diagnosis until time of study** **(caregiving experience) (years)**	
2–3 years	5 (50)
4–5 years	4 (40)
5+ years	1 (10)

a Median (min, max).

## Themes

**Figure 2:**
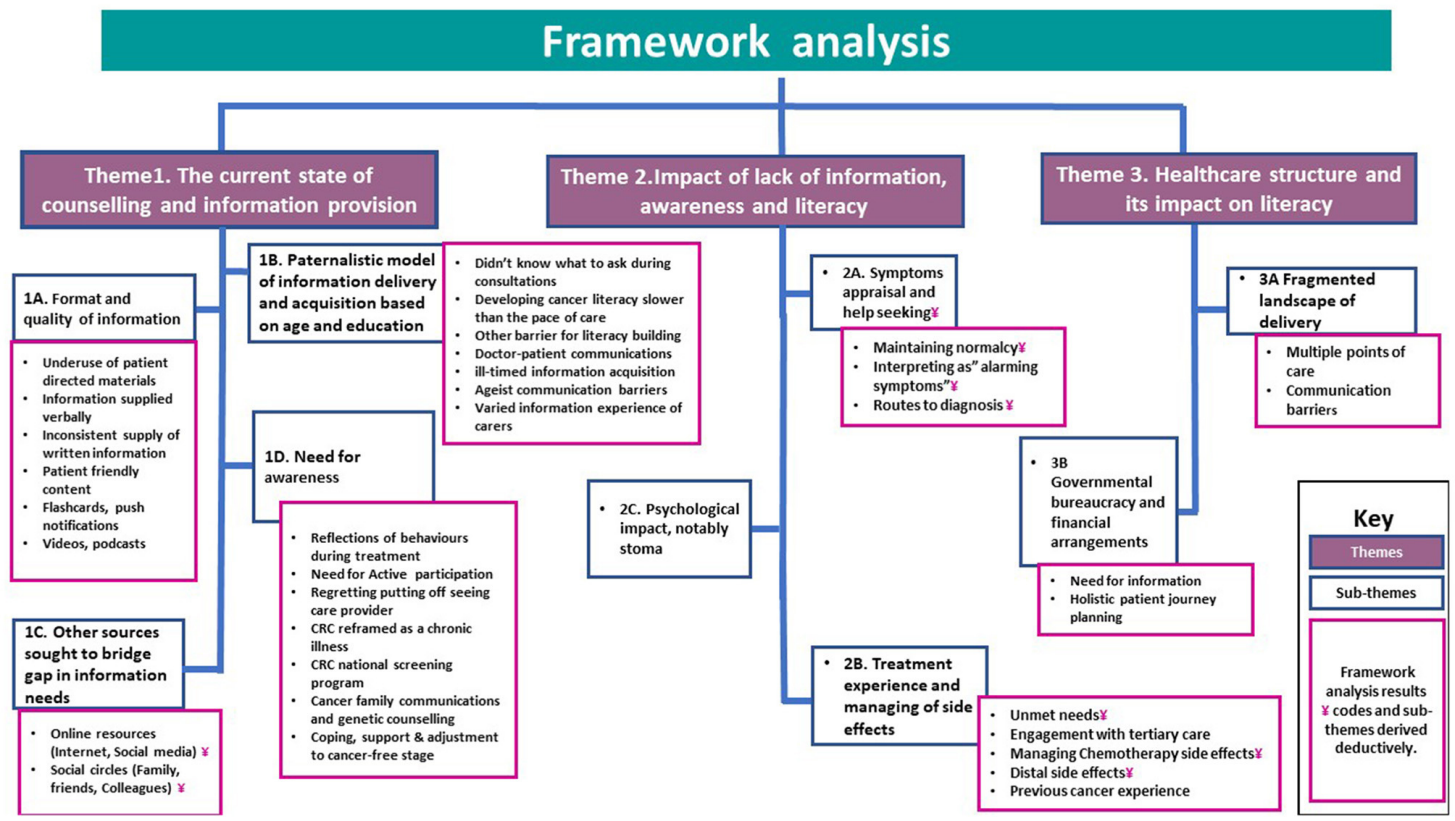
Theme(s), subthemes, and framework analysis findings.

The Thematic analysis revolved around a central concept of “exploring literacy and its impact” and three overarching themes: *(1) Current state of information provision and counseling, (2) Impact of lack of information, awareness and literacy, and (3) Healthcare system structure and its impact on literacy*. Themes, subthemes, and framework analysis findings are depicted in Figure 2. Additional comprehensive list of quotes that support the finding is found in Supplementary material 4.

### Theme 1. Current state of information provision and counseling

Subthemes (1A−1D) depict the multifaceted and contextual interactions with the health care system that influence literacy development, information acquisition, and processing.

#### 1A. Quality and format of information

Underuse of patient-directed materials, information presented verbally, and inconsistent written materials across departments/facilities, led to inadequate cancer-related HL. Patient counseling was only delivered verbally during doctor-patient interactions, and patient handouts differed widely among departments and institutions where patients were treated. Patients at various hospitals and stages complained about inconsistent patient-directed materials. In addition, booklets and written counseling plans were not matched to respondents' requirements or stage of therapy. Respondents said that while they are provided in-depth information regarding their condition, it is written in language that they cannot comprehend, with the latter acting as another barrier to successful counseling and HL development.

“*At the other hospital where I received chemotherapy, no patient counseling or explanation of my medications or side effects is provided; you simply get the pills and nothing more.” (CRC Survivor male, 58 years)*

The sub-quality of information extended to dietary education and guidance.

“*I received nutritional plans from the center, like I should avoid legumes, but there is no specific plant, or food, they said you should try and see which types of foods work best, but I had many problems, like bursting, filling quickly, bad smell.” (CRC survivor male, 63 years)*

Even after becoming cancer free, one respondent reflected on the dire state of resources and information. The interviewee underlined the need for trustworthy, patient-directed resources to assist them cope, support, and transition to cancer-free stage since they didn't know where to turn for credible information regarding CRC.

“*Finding what I need might be difficult. Since beating cancer, I've realized how much I still need to learn, but I don't know where to seek for resources*.” (*CRC survivor male, 58 years)*

#### 1B. Paternalistic model of information delivery and acquisition based on age and education

Mistimed or withheld information, lack of confidence in asking questions or understanding what to ask during consultations, ageist communication, and information provision that is dependent on the receiver's education level were all examples of paternalistic information flow and communication between healthcare practitioners, patients, and their ICs, as well as between ICs and patients. All these approaches lead to poor HL or HL that grows at a slower rate than care delivery. These communication approaches can undermine patient empowerment and participation in treatment decisions. Some patients indicated they didn't know what to ask during diagnosis consultations, but having all of their medical data and regular follow-ups let them feel more confident about information sufficiency. Others stated that their doctors pay great attention to them, but that it is up to the patients to ask the correct questions in order to receive the best answers, which is tough and leaves patients with a shallow understanding of the disease and many unknowns.

“*All of my queries were answered by the physicians and nurses, who were very kind and helpful. But I'm not always sure what questions to ask to get a deeper understanding……” (CRC survivor male, 58 years)*

“*I honestly don't know, since they do investigations and tell me they are doing this and that. I didn't choose which therapy to do. I just go to my doctor, and he tells me what to do. After four cycles, I'm not sure why the doctor recommended a colonoscopy.” (CRC survivor male, 72-years)*

The treating physicians' knowledge, reputation, and faith influenced patients' paternalistic decision-making styles. Though finding the right specialist was difficult, patients feel safe in their care. One cancer survivor said he let the doctor make all treatment decisions due to his urgency and lack of cancer knowledge. After treatment, he realized his lack of plan engagement hampered his post-treatment management. Participants who did not actively participate in decision-making and placed too much reliance on their doctors acknowledged regretting these behaviors, but they also confessed that they had attempted to educate themselves but failed mostly due to lack of resources. “*I didn't have much to do with my treatments, and I mostly relied on the treating surgeon. After all, it's all about having a good surgery, and you don't send a boy to do a man's job.” (CRC survivor male, 72 years, high school)*

“*The consultant is a well-known surgeon who has done many similar surgeries, so I knew I could trust him. He was in charge of everything, along with the medical teams. Now it's up to me to take care of myself, which is why I started learning about my symptoms. I didn't find many helpful things on the internet, like diet plans for people with colorectal cancer based on their stage and procedure.” (CRC survivor male, 58 years)*

Some patients thought herbal, traditional, or alternative medicine could help them heal, but they were concerned their doctors would patronize them because they solely believed in conventional therapy.

“*Doctors are somewhat patronizing, and sometimes they just push conventional medicine, but I think I can find more ways to help me heal and boost my immune system. Cancer therapies have a lot of toxicities that hurt your body, so I'm tired of the side effects, and if I tell my doctor that I'm taking soursop, turmeric, or I'm cleaning my colon, he or she will look at me like I'm crazy.” (CRC survivor female, 61 years)*

In addition to the healthcare team's paternalistic approach to patient information, some ICs used ageist approaches to seek age-biased professional decision-making about patient diagnoses and treatments. Caregivers felt that divulging the diagnosis would cause emotional distress or lower the spirits of the patients they care for, especially elderly, disabled, or advanced disease patients. Some patients were informed unwittingly or late in their treatment. ICs also stressed the importance of presenting information in a way that never caused anxiety and was patient-friendly, personalized, and easy to understand because patients' emotional and cognitive capacities may hinder their ability to learn during therapy.

“*We were worried about the emotional and mental reactions, so we talked to the doctor and the whole staff to not tell him [his 71-year-old dad] anything. However, a doctor who wasn't on staff got into his room and told him everything, and my dad thought she was mistaken for another patient because she didn't know about our agreement to keep it a secret. We needed a psychiatrist to inform him professionally. Our biggest concern was that telling him would kill his positive attitude. Seniors fear everything*.” *(IC male, 26 years)*

Depending on their education, ICs received different types of information, which led to a variety of experiences. Caregivers with a medical background reported being well-informed about the patients they were caring for and that doctors included them completely in decision-making. Additionally, their education enabled them to find reliable information. Despite being an engineer, one carer said his education didn't help him find relevant CRC information.

“*I am an engineer, and I had no idea what cancer was or what was going on with my father.” I kept searching and reading until I discovered that the colon is 1.50 cm long, which I had never known before.” (IC male, 26 years)*

“*The consultant involved me in every aspect of my dad's treatment, even the protocols, because I'm a doctor. I also used medical resources to learn about his case and prognosis. It's different when you explain these things to carers, who are usually more engaged if motivated or have higher education.” (IC female, 27 years)*

#### 1C. Other sources of information sought to bridge gap in information needs

Participants preferred patient-friendly videos, animation, push notifications, and podcasts. One patient was interested in reading extensively about the illness. Alternative information sources and patient experiences are listed below to fill the information gap.

##### Online resources

Several patients who used the Internet to fill their knowledge gaps during treatment said they learned more about cancer by reading online cancer-related material. Several informants said they had trouble finding useful, relevant information to improve their literacy. Some survivors gave generic or non-specific answers when asked about their search strategies. Few patients' search strategies aligned with their treatment plans, so their searches were successful. “*I specifically researched the “FOLFOX‘” chemotherapy regimen online. Although my experience was terrible, the doctors never addressed these issues, so I researched how to prepare for chemotherapy. I mostly use the internet for diet and lifestyle research.” (CRC survivor male, 68 years)*

Patients' ability to access complex and in-depth information online about some problems, such as LARS (Lower anterior resection syndrome), was limited due to lack of information and inadequate education catered to their HL by their clinicians.

“*I won't be able to get the finest information if I don't know what exactly is wrong with me and what physicians label it in their terminology because they frequently withhold information regarding anticipated symptoms until they manifest.” (CRC survivor male, 67 years)*

Lack of understanding of colonoscopy findings and the desire for more information prompted the use of social media to bridge information gaps; however, the downsides included misinformation, language barriers, and emotional and psychological ramifications for some patients.

“*In the Facebook group (ask doctor), people share treatment reports and ask doctors to explain colonoscopy results. I tried to learn more because I didn't understand my case after initial consultations…. I was shocked when the admin said anything cancer-related should be discussed privately between the patient and his provider… I knew “it's cancer” before meeting the oncologist.” (CRC survivor male, 68 years)*

##### Patients' social circles

Patients relied on family and friends as mediators with medical expertise or digital abilities to help them improve their literacy by deciphering medical records, communicating with health specialists, and seeking information.

“*When I'm with my stepmother at her appointments, she feels more at ease. Sometimes she's too shy to tell her doctor what she's going through with her rectal cancer, so I talk to their doctors about these symptoms.” (IC female, 41 years)*

Lifelong ostomates valued peer support in the form of practical and psychological advice. “*Because it was new [ostomy bag], I was upset, but a co-worker with a similar issue helped me keep going. He had a permanent colostomy before me. He gave me practical advice on how to live after the colostomy and informed me of his subsequent actions.” (CRC survivor male, 58 years)*

#### 1D. Need for awareness

CRC was reframed as a chronic illness, and participants recognize the need for a national screening program and cancer family communications and genetic counseling information. Some patients stressed the importance of a CRC awareness and screening program, “*Neither of my parents work in medicine, so they don't know about colonoscopies. Both my parents never had colonoscopies. I told them because they're over 50.”* (IC female, 27 years). Whereas, others regretted delaying medical treatment due to ignorance and lack of HL. “*I didn't even ask why my blood level is six or what it means. This could be because I don't fully understand what it means.”* (*CRC survivor female, 33 years)*

Younger survivors with children described how they sought information to help them explain the importance of cancer to their children. “*Googled “how to tell my kids I have cancer…the internet helped, but not enough. Cancer is hard to say. Fearful. Cancer frightens. When I got home, my kids saw my scars and colostomy bag. That day, their innocence was taken from them. They grew up when life suddenly became real. I don't want them to wake up one day and say, ‘I wish I hadn't done that,’ so I think it's inevitable. ‘Mom, you got cancer? Returning?’ I struggle to balance my patient concerns with my motherly duties.” (CRC survivor female, 36 years)*.

ICs, in particular, stressed the importance of cancer family communications and the need for resources on how to communicate cancer-related topics to patients in a way that preserves their spirits and provides psychological support. They also reported that it was challenging to explain genetic counseling and educate family members. “*I'll never forget the doctor's words to me after the surgery: ‘It may happen to you and your siblings and sisters.’” Therefore, it is hereditary, and we need to monitor…. my brothers had phobias…. they wished to know when they should be required to observe, conduct exams, and so on. When must screenings be conducted if there is a program that informs people, particularly those who have a family history.” (IC male, 35 years)*

### Theme 2: Impact of lack of information, awareness and literacy

Literacy, awareness, and information availability had an influence on CRC patients' symptom assessment and medical help seeking. In addition, it impacted CRC patients' treatment experiences and quality of life including self-efficacy in managing treatment side effects and the psychological impact of treatment, particularly stoma.

#### 2A. Symptoms appraisal and medical help seeking

Patients sought medical care in diverse ways due to the lack of standardization of health-care pathways. Symptoms appraisal and help seeking in the pre-diagnostic phase outlined diagnostic routes used and symptoms evaluation and interpretation whereby participants pondered on physical changes that they later identified as symptoms. The timing, consistency, and frequency of bowel movements were examined and compared to normal or anticipated patterns. Initially, symptoms were normalized and understood as innocuous attributions. Patients with chronic stomach pains ascribed their symptoms to H-pylori, whereas those with occasional constipation or regular diarrhea believed they had irritable bowel syndrome. A change was more likely to be recognized as abnormal if it was large, emerged quickly, became recurring or was accompanied by additional changes, lasted longer than expected, made routine daily activities difficult, or raised concern among family members. “*My father lost 10 to 11 kilos in 2 weeks while doing the same activities and eating the same diet; nothing had changed except for the rapid loss of weight, and once we received the blood test results, we knew that his Hb is 7.5 or 7.8, we freaked out…. It would be impossible for us to tell if my father has cancer without any symptoms of weight loss.” (IC male, 26 years)*

Participants who thought their bleeding was due to hemorrhoids were likewise unlikely to contemplate CRC, but those who observed that it persisted were more likely to consider cancer or seek medical attention. “*Because there was bleeding from the anus, I initially assumed I had hemorrhoids for like 2 months but after that when I used to go to the restroom, I would notice that the blood had darkened. When I use toilet paper, I notice that there is a bulk and some hardness in the anal area. So, I went to the hospital ER.” (CRC survivor male, 58 years)*

Despite significant changes in bowel habits and despite the advice of his family, one patient managed to maintain some feeling of normalcy while adjusting to a severe and debilitating condition. This is due to the fact that he assumed that as he had a clear medical history, his digestive function was gradually declining with age. “*I used to go to the toilet to poop every 3–4 weeks recently, but before that I remember I used to go more often, I thought it was normal, I was oblivious about that …… My daughter insisted I be checked out since I had restroom problems. I was obstinate and didn't want to listen to anyone's advice…. In my mind, it seemed sense that as we grow older, our bodily functions would slow down….” (CRC survivor male, 72 years)*

For other patients, preserving normalcy included self-medication with laxatives or antidiarrheals for symptomatic relief utilizing herbal or traditional remedies. However, in cases where the physical effect of symptoms became intolerable or threatened regular daily activities, patients were more likely to seek medical attention. “*I experienced constipation for 10 days, tried senna, and lactulose from the pharmacy, but too full and unable to defecate. I visited the ER because I couldn't sleep or sit and had stomach pain.” (CRC survivor male, 58 years)*

Fainting, dizziness, bloating, and fatigue are examples of non-specific changes that made some patients being oblivious of the implications of their symptoms. Most of the time, vomiting or anemia were not thought to be signs of CRC. Hence, participants took vitamins and minerals to alleviate these negative effects. The rationalization of pre-existing assumptions, such as they don't have a cancer family history, broadened and validated their feeling of normalcy. Participants, particularly those younger in age, rationalize their symptoms by asserting that they had considered cancer or CRC as a disease that affects the elderly. Additionally, younger patients said they were too busy with careers and families to seek medical attention when they first experienced vague symptoms, so they never considered CRC a diagnosis. “*It seemed exceedingly unlikely that I would be given a cancer diagnosis at my age, even if I was completely oblivious of what are the symptoms.” (CRC female, 36 years)*

While some participants found it helpful to talk to family and friends, this was not always the case, and other participants disregarded their relatives' suggestion to see a doctor. However, one survivor said that her loved ones discouraged her from seeking medical attention in favor of more natural, holistic approaches. “*Friends and relatives assured me that my constipation was typical and that I shouldn't worry about it, suggesting various natural remedies and herbs.” (CRC survivor female, 61 years)*

One participant, as an exception, was able to spot the changes in bowel movement right away since she had previously experienced regular bowel movements and who, on the advice of her family decided to seek care in the private sector. “*I rarely get constipated because I eat a healthy Mediterranean diet and go to the bathroom in the morning or after coffee. After a month, a drugstore laxative didn't work, so I was scared… My husband called a doctor cousin after I told him. I was moved to this tertiary hospital after a private colonoscopy a week earlier.” (CRC survivor female, 59 years)*

Five out of 15 patients were diagnosed at the emergency department (ER) as were patients with a non-specific or non-classical presentation, or those with rectal bleeding. “*I was at a wedding when I frequently felt like vomiting. They called an ambulance to take me to the emergency room, where I had a CT scan and lab work.” (CRC survivor male, 72 years). Three* out of fifteen were self-directed to the private sector based on family consultation or advice. After developing a symptom, some respondents sought care from multiple physicians, switching back and forth until their symptoms deteriorated. Because of the delay in diagnosis, several patients needed to be admitted as emergencies when their diseases deteriorated after having been treated for another diagnosis. One interviewee described how general practitioners misinterpreted her symptoms and treated her until her condition deteriorated. “*I couldn't eat or drink because I felt sick and lightheaded. Constipation prevented me from pooping seven times in 2 months. The private hospital doctor near my home didn't examine me thoroughly because he thought my problem was minor. However, I was prescribed medication for symptoms relief. When my condition deteriorated, my spouse took me to this hospital, where I was finally diagnosed.” (CRC survivor female, 53 years)*

#### 2B. Treatment experience and managing side effects

CRC survivors and ICs cited numerous unmet needs associated with coping with adverse effects of chemotherapy or post-operative care activities. The latter was mainly in relation to ostomies, for example difficulties in locating adequate ostomy supplies, affording the expenses of stoma supplies in addition to dealing with other stoma-related issues such as leakage, poor odor, herniation, prolapse and psychological-practical ostomy life style adjustments. “*Because of the colostomy, I can't sit in a chair for long periods of time without experiencing pain on the surgical side. I had to wear dishdashi (long dress) at first since I was unable to wear trousers or my own clothing. In the end, I figured out how to dress regularly again, including using a belt to secure it…. I can't even wear my socks without the help of my wife. The transition to my unfamiliar setting was taxing on my body and mind, and it took me nearly 1 year and a half to adjust.” (CRC survivor male, 58 years)*

The social stigma attached to discussing bowel abnormalities and ostomies were also raised by interviewees. Because they perceived their stoma as a short-term fix, they lacked self-management skills. Several ostomates had a low quality of life as a result of their stoma. Conversely, those who viewed it as a permanent component were better able to mentally adjust. Several responders said they weren't given nutritional guidance or time to prepare for treatment or colostomy management. “*When the doctor said the stoma is “temporary,” I didn't worry about having it for a short time and didn't pay much attention to what does it mean to have a stoma!”* (*CRC survivor male, 53 years)*

Despite their low quality of life, frequent hospitalizations, and ER visits, participants believed they should accept chemotherapy's side effects because of their preconceived notions about their inevitability. Because alopecia is a common side effect of chemotherapy, some patients reported only questioning their doctors about it. However, some informants experienced distal side effects that were difficult to manage and differed from their expectations. “*They gave me chemotherapy, and they said it won't cause any hair loss, this is what I was concerned about, but now I have numbness in my feet that is getting worse…. “(CRC survivor male, 56 years)*

Several respondents said they had a dreadful experience with side effects and that they had unexpected side effects that their physicians hadn't warned them about. Some patients felt misinformed because their assumptions about treatments and potential adverse effects did not match what their doctors had told them. As a result, they had frequent emergency hospitalizations. “*It has been a hell of a ride… The five rounds of chemotherapy were a nightmare and absolutely destroyed me, in contrast to what they had told me…*… *I had terrible diarrhea and had lost a lot of weight, so I stayed there for 3 or 4 days. I used to weigh 75 kg, but when I was admitted, I only weighed 59 Kg, so I was treated as if I weighed 59 kgs until my immunity came back to normal, which was zero.” (CRC survivor male, 58 years)*

On the other hand, patients or caregivers with prior cancer caregiving experience reported more resilience, developing knowledge and skills and acceptance of the condition, despite the challenging experience they had, which enabled them to better cope with the disease. “*Historically, both my aunt and uncle (on my dad's side) and my aunt (on my mom's side) died from cancer. My mom had two types of cancer, while my dad had it three times and is colon-free (nearly total colectomy). We're familiar with ‘cancer.’ We all get regular check-ups but prepare for the worst.” (IC female, 38 years)*

#### 2C. Psychological impact, notably stoma-related

Psychological barriers to professional care (guilt, embarrassment) and post-surgery weight loss among ostomates make life with an ostomy less than desirable and keeping ostomates at home and away from social engagements. As mentioned above, patients and ICs felt the pre- and post-operative food instructions were inadequate, resulting in poor self-management. Some patients lost a lot of weight because they couldn't change their diet or choose foods that wouldn't overfill or leak the ostomy bag. Others felt ashamed and struggled with stomas. “*I was ashamed because no matter what I ate, it would fill up and burst. I lost my appetite and couldn't eat a variety of meals after having a colostomy, so I dropped from 85 to 60 kilograms.” (CRC survivor male, 53 years)*

### Theme 3: Healthcare structure and its influence on literacy

The fragmented healthcare system, government bureaucracy, and financial arrangements affect cancer patients' ability to learn about and discuss their condition. Most Jordanian cancer patients receive treatment at various government-funded sites. Fragmentation and the need to acquire financial arrangements and government cancer insurance information before sectoral transfer for treatment fulfillment made government formalities time-consuming and burdensome. This calls for a holistic journey strategy and process simplification to reduce red tape. “*Sometimes I don't even know which care provider is taking care of me ……When they moved me from one facility to another, I also had to fill out government paperwork and applications.” (CRC survivor male, 58 years)*

Without interoperable electronic health records and synchronous and asynchronous patient notes, specialists from different health systems are less likely to communicate electronically (such as tumor boards). Thus, fragmentation may decrease care quality and increase inequality. “*My doctor, a medical oncologist at another facility, doesn't see me regularly. So, I go every 6 months. My doctor won't tell me when to see him or her. The hospital doesn't care if the patient shows up… Never…”* (*CRC survivor male, 58 years)*. Some cancer patients had to be readmitted to hospitals for treatment multiple times because of poor self-management, communication hurdles, and the fragmented nature of cancer care. “*I didn't know who to call when I had side effects from chemotherapy because I got it at a different place not this hospital. So, my family had to pick me up again to the ER.” (CRC survivor male, 58 years)*

## Discussion

Using qualitative approaches, this study moves beyond the limiting definition of HL toward a broader conceptualization that accounts for the complex nature of HL phenomena through an in-depth analysis of the experiences of CRC survivors and ICs during their interactions with the health care system along the care trajectory from pre-diagnosis to survivorship. The study highlighted contextual, individual, sociocultural, and healthcare system determinants affecting HL of survivors and carers and information acquisition, provision and interpretation. In addition, it highlights the impact of HL on symptoms interpretation and seeking medical care and resources used to bridge this gap, while outlining changes that are needed to improve health outcomes.

### Internal/external healthcare system context and HL status

Due to methodological inadequacies and varied HL conceptualizations, Humphrys et al. (48), could not fully analyse HL's impact on early cancer diagnosis. However, low HL is linked to poor quality of life after cancer diagnosis, treatment decision-making difficulties, and low cancer screening rates.

According to the findings, the environment and organization of the healthcare system had an impact on how cancer survivors with CRCs and their carers were developing their cancer literacy. Many CRC survivors in Jordan seek emergency or tertiary treatment due to a lack of awareness of CRC symptoms, delayed help-seeking, and unstructured health care and referral pathways. The experiences of those patients who received a cancer diagnosis through an emergency pathway indicate that the majority of them had recurrent evaluation and help-seeking (49). These findings concur with Abu-Helalah et al. (50), who showed that the most common causes of delayed presentation among Jordanian CRC patients were misdiagnosis by physicians or pharmacists (38.4%), a lack of understanding that the patient's symptoms were suggestive of cancer (58.5%), and a lack of motivation to see a doctor (3.0%). The findings demonstrated a knowledge gap about CRC screening and symptoms, emphasizing the need for Jordanians to adopt improved health care strategies and CRC promotion efforts. Lack of patient-centered training programs on the importance of CRC screening as well as insufficient CRC publicity may be to blame (51, 52). These results support earlier quantitative studies that called for a countrywide deployment of a CRC screening program, suggesting that as a first step, this CRC screening program should be adopted at primary care clinics and community hospitals at a national level. This qualitative sample, although is supportive of this argument, this goes beyond the scope of this research. The complexity of health care systems, especially in cancer, raises difficulties for patients and users to navigate them during the pre-diagnostic period (13, 53). HL-NAV is needed for healthcare system navigation and communication. To navigate the health care system and find the best solution for their health issues, patients must be able to choose an appropriate access point, explore many institutions, and identify an appropriate entry point (54, 55). HL-NAV is relational and depends on patients' HL-NAV and the health care system's complexity and demands, particularly the quality and types of information and communication available (56). This study highlighted low HL-NAV among CRC patients driven by a bureaucratic and fragmented healthcare system and lack of effective information and resources. Low HL-NAV among patients results in confusion, useless and unpleasant searches, ambiguity, and treatment gaps as seen in the results (57). In the digital era, developing digital solutions to help patients and carers navigate the healthcare system is vital to overcome the fragmented landscape of care delivery, governmental care centrality, and financial arrangements that prevented some survivors and ICs from getting care. Future CRC diagnosis will increasingly incorporate digital technology. Smartphone symptom-checking apps are available in several countries (58). These apps collect gastrointestinal symptoms to track pre-existing disorders. These programs can instantly update the patient's electronic health records (EHR). Thus, detecting delays and investing in digital solutions to get patients to the correct specialist at the right time can improve CRC diagnosis. Although CRC was reframed as a chronic illness, which emphasizes the need to focus on survivorship care, recent research by Melhem et al. (4), demonstrated that CRC survivors have persistent unmet information needs during this stage. Therefore, CRC survivors and ICs sought alternate sources of information to bridge their informational gaps and improve HL *via* the internet and social media, but their benefits were offset by lack of DHL skills, inadequate search strategy, and suboptimal online information quality (15). Furthermore, survivors sought emotional and informational support from family, friends, and colleagues. Due to a lack of HL and standardized information, several patients showed low stoma self-efficacy and stoma-related psychological impacts, such as feelings of shame or humiliation. Pate et al. (59) reported that pre-surgery education and standardized, health-literate written materials improved stoma self-efficacy. Therefore, by strengthening self-efficacy, patients may be better able to manage their ostomy and care for themselves after leaving the hospital, thus avoiding issues and improving outcomes.

A growing body of research reveals that HL is contingent on the individual's abilities and skills as well as the requirements and constraints of the healthcare system (9, 36, 60). Despite rising expectations, cancer survivors struggle to manage their disease and care (61).

Cancer literacy was influenced by organizational literacy contextual factors, including access and verbal/written communication which were substandard and inconsistent in addition to the format and quality of information provided that were suboptimal and not tailored to CRC survivors' HL skills and information requirements. The information was in jargon and not formulated in patient friendly formats which were deemed more favorable such as videos, podcasts, animations of push notifications to match patients and IC HL skills. Varied experiences in engagement in information provision and decision making and involvement in treatment calls for the customization of patient/carers information delivery that is adaptable to the HL level. This will ensure health equity in information and care delivery without exacerbating inequalities among CRC survivors and ICs.

OHL is a new concept that emerged to satisfy the requirements of the majority of patients with poor HL (60). It refers to a healthcare delivery system that uses strategies to assist people in participating in their treatment, navigating the healthcare system, understanding medical information, and taking charge of their health (60, 62, 63). The crucial function that OHL plays in improving patients' self-management support and communication have been underappreciated (64, 65). Consequently, healthcare reforms necessitate the development of responsive health care delivery systems and health-literate organizations that incorporate HL into their strategies (60). To the same extent, there is a need to give solutions to alleviate shortages and obstacles of HL by helping patients better comprehend health information, simplifying health care, and receiving more comprehensive support (66). Improvements in HL are necessary before patients may be empowered, which would transfer authority and responsibility from healthcare providers to patients and boost patient engagement. This transition may increase the quality of care through improved treatment decision-making and the utilization of self-management opportunities, resulting in better health outcomes (67, 68). Enhancing HL by fostering open lines of communication and collaboration between healthcare providers, patients, and family members can expedite the delivery of high-quality care that is both personalized and cost-effective. In order to facilitate the shift to health-literate organizations (60), it is necessary to design patient-centered literacy programs that target and focus on patients with lower levels of literacy.

The notion of OHL emphasizes the challenges each patient encounters over the course of treatment and it can only be understood in the organizational context of care since patients' capacity to absorb health information and navigate the care-seeking process is tied to healthcare system needs and the challenges they experience (60). There are several approaches for organizations and services to promote HL. Design elements that make navigation easier for patients and their caregivers can be used in health clinics and hospitals. The signage at hospitals might be written in simple language so that individuals of all literacy levels could read it. Through simple-to-use internet platforms, new technological advancements can make it easier to acquire healthcare services.

### CRC patients and carers' health care interactions and HL

The ability to make informed decisions is constrained by a lack of relevant knowledge, the complexities of benefits and drawbacks of therapies, and uncertainty pertaining to the care process (66). However, the urgency in decision making prompted inactive involvement of some CRC survivors while delegating decisions to their oncologists. CRC patients do have preferences regarding different treatment options and outcomes, however, these preferences are not homogenous and seem to depend on personal factors like age and gender. Despite the existence of these preferences, the majority of patients prefer a passive role in the decision-making process, which in part may be explained by the severity of the disease (69). Interaction with the healthcare system was influenced by ageism and medical paternalism which may impede the development of cancer related HL. Physicians' decision-making processes involving information, communication, and treatment have been found to vary depending on the patient's age. Many cultures, especially those of Asia (70, 71), Southern Europe (71, 72), and Latin America, are reluctant to inform elderly patients of a cancer diagnosis or prognosis, which echoes the findings presented. One study indicated that physicians spent less time with older patients and paid less attention to their needs and choices compared to younger patients (71). Cancer patients in Jordan and culturally equivalent nations favor paternalism, if not authoritarianism, when it comes to medical decisions. Patients relinquish their autonomy because 'the doctor knows best' (73). Lack of confidence, ambiguity about which option to take, competing ambitions, or anticipating self-blame for disappointing results lead to this mindset (74). Paternalism remains the most preferred source for information provision among CRC survivors despite Jordanian physicians' endorsement of patient autonomy, engagement in treatment options and self-management (15). This runs counter to the assumption that paternalism should be avoided. However, this notion should be tailored to cultural perspectives and attitudes that cannot be changed overnight (15, 74). Treatment-survivorship expectations gaps were mentioned by some CRC survivors due to their passivity and late engagement in their care which undermined their ability to develop HL skills and knowledge to effectively self-manage post-treatment. Several studies on CRC survivors highlighted the dissatisfaction in HCP communication styles and lack of knowledge, time or empathy (31, 33). Other survivors' related factors for miscommunication include embarrassment to ask providers for support, for fear of appearing ungrateful, bothering the HCP or making a big deal out of symptoms that may be normal (6), a finding echoed in this study.

The relationship between HL and empowerment is inconclusive. The WHO recognizes HL as a social determinant of health and improved HL as a key goal of public health. Increased HL is identified as a necessary prerequisite for achieving patient empowerment, which can reduce the utilization of health services and healthcare costs (19). On the other hand, a review by Schulz and Nakamoto (75) highlighted that despite the effects of HL and patient empowerment being intricately intertwined, the two concepts are independent and distinct. HL does not always imply empowerment and *vice versa*. Mismatches between the two can have negative consequences. High HL without a corresponding high degree of patient empowerment may create unnecessary patient dependence on health professionals. However, both are important patient-related variables to consider during screening and health promotion campaigns for the general population (75, 76).

## Strengths and limitations of the study

To the best of our knowledge, this is the first exploratory study to focus on CRC survivors and ICs healthcare experiences and its relationship to the multidimensionality of HL. The research conducted in Jordan; a country representative of Arab Middle Eastern culture also illuminated the importance of understanding the sociocultural factors impact on HL in the context of oncology. The study's strength rests in its in-depth interview methodology and open-ended format. Participants were able to bring up themes and subjects that were important and significant to them, which may not have come up in structured interviews or research that used quantitative methods. Although HL in its restrictive skill based and functional view can be measured using standardized assessment tools, the qualitative methodology allowed the understanding of survivors and ICs health experiences and their relationship to the broader sense of HL as crucial element for patient engagement. It also allowed the linkage of these aspects to the multiple dimensions of HL (Nav-HL, OHL, and DHL). The findings of the study maybe be used to design an e-health teaching program for patients with poor literacy and guide healthcare services reforms for this population. Additionally, our results highlight the need of changing the Jordanian health care system into a literate system.

Convenience sampling from one large semi-government-run tertiary hospital reduces the generalizability and may be a limitation of the study. However, individuals were treated in a variety of settings in multiple facilities, since their therapies were carried out in a number of hospitals in private and public sectors, thus their aggregate experiences may be holistic and provide insight into a population with limited research.

### Implications for practice and future research directions

Low Cancer HL is a barrier to efficient and timely diagnosis and care delivery across the cancer continuum. Empowering cancer patients is essential for better outcomes. The need for adopting healthcare policies to transform healthcare organizations into “literate organizations” that provide health practitioners with education, information, and tools, need to be addressed by healthcare policy maker to enhance cancer literacy. In order to guarantee that the patients receive timely and adequate care, public health policy should also consider the establishment of national screening program and awareness through online educational programs. These programs must be designed to raise patients' comprehension of their diseases and treatment alternatives, which should result in patients who are more knowledgeable and empowered.

## Data availability statement

The original contributions presented in the study are included in the article/Supplementary material, further inquiries can be directed to the corresponding author.

## Ethics statement

This study was authorized by Kingston University's ethical guidelines for scientific research (approval number/1416) and the Internal Review Board (IRB) at Jordan University Hospital (JUH), protocol ID (10/2019/8990). The patients/participants provided their written informed consent to participate in this study.

## Author contributions

SJM and RK conceived, designed, and planned the study, did proofreading and editing, and conducted data analysis and interpretation. SJM did data gathering and translation and wrote the substantial amount of the article. RK being a senior author supervised the implementation of the Project. SN-G contributed to linguistic validation of transcripts, codes, and themes mapping. All authors has been approved and revised the papers.
