# Supplementary material for: Cancer literacy among Jordanian colorectal cancer survivors and informal carers: Qualitative explorations
Source: Front Public Health. 2023 Mar 20;11:1116882. doi: 10.3389/fpubh.2023.1116882 (PMC10067669; doi:10.3389/fpubh.2023.1116882)
Supplement: Supplementary file 1 [file Table_1.DOCX]

**Interview guide**

**Part 1: Interviews with Colorectal Cancer Survivors**

| 1. **Firstly, I would like to talk about your *journey with cancer* from time of diagnosis.**    1. Tell me about your journey with cancer, how it was diagnosed, how was the treatment process.   Useful probes: tell me more? Why? Explain to me? Can you give me an example? Give me a picture, a description of the whole process, how it goes?  1.1.2. Tell me about your experience (challenges difficulties, struggle) with cancer? (Individual level, family, job-related, society, ask about different stages, use probes)  1.1.3. Let’s talk about the treatment; tell me more about the counselling process? How you were counselled about the medications, treatment plans & treatment side effects?   - 1. **I’d like you to share your experiences on any obstacles during your treatment process? How were your concerns being dealt with?**       1. How did you feel about the adequacy & quality of information you received from the health teams? Were there any communication barriers? What have you done to overcome any barriers to access health services?   2. Did you have any particular concerns about the treatment side effects (Y/N)? Were your concerns fully addressed?   Prompts: how well did you understand the information supplied? Did you get any written information, like leaflets or informational booklets? what resources were needed and not supplied   - - 1. how did you deal with any treatment-related adverse events post-discharge?   1. **Let’s focus on the follow up treatment. Now you are visiting your doctor for a follow up, tell me about your typical journey? How does it usually go? if follow up is completed, how long did it take you until you finished your follow up?**      1. Can you describe the typical activities you do in the process? Which activities take the longest waiting times?   *Prompts*: did you know what to expect? Did you know the follow up plan, appointments, treatments, tests? What were the challenges of this period? What coping mechanisms or support strategies did you use? |
| --- |

| 2. **Communications with Healthcare professionals** |
| --- |
| - 1. **Focusing on communication, tell me how you feel about the communication with the medical team? Which form of communication was mostly used? For example, face to face, phone e-consultation, chatting, video consultation? Was communication consistent in quality and frequency throughout the treatment cycle and follow up?**       1. do you feel included in treatment plan? What were the most information needed/desired during those communications/consultations? Prompt: Can you tell me why?      2. Is there anything you feel can be improved or anything to be recommended that you would like to share? |
| 1. **Coping strategies** |

| - 1. **Focusing more on coping strategies, what are/were your coping/support strategies to combat the disease during or after completing your treatment?**   2. **How did you confronted the challenges (body image, emotional adjustment, spiritually, networking, role of religion, extent of changed identity, relationships with others?**   **3.2. What did others close to you do for you that made a difference?**  **3.3. Did you join a support group? If so, how did it help? If not, why?**  **3.4. Were there any online (apps, websites)/offline programs or services offered to you or you used as support or information during the treatment process? Were they helpful? What other support would you have liked**?  *Probes: Give me an example?*  *If online services were available/used, give me examples? Give me examples of alternative online resources you used to help you in your battle with cancer?*  *If any useful resource (like a website or mobile app) is available, would you use it?*  **4.Is there anything else you would like to share with us on the topic?** |
| --- |
