# Supplementary material for: Cancer literacy among Jordanian colorectal cancer survivors and informal carers: Qualitative explorations
Source: Front Public Health. 2023 Mar 20;11:1116882. doi: 10.3389/fpubh.2023.1116882 (PMC10067669; doi:10.3389/fpubh.2023.1116882)
Supplement: Supplementary file 2 [file Table_2.DOCX]

**Topic guide informal caregivers focus groups**

**Part 2: Focus groups interview schedules with informal caregivers**

**This part aims to determine the potential features of such an application in light of your preferences, perspectives and experiences.**

**1.Tell me about your experience of using mobile applications? what type of apps do you use for personal and professional use? What about health/ fitness/ medication reminders/nutrition apps?**

**2.. In your opinion, what is the role and importance of mobile apps in supporting cancer patients and their informal caregivers?**

2.1. What are the benefits, advantages of such a digital solution as a supportive tool for you as a caregiver through the patient journey and for the treatment plan?

**3. What would you like this app to offer**

3.1. What information would you like the app to have as a caregiver? (prompts: information about short and long side effects, how to manage between cycles, nutritional plans, effect of cancer on relationships, insurance, appointments, follow up schedules (mammogram, ultra-sound, colposcopy etc.)

3.2. What other features would you desire?

(prompts communication tool with HCP, side effect alert, medication reminder, online consultations? Communication with HCPs?).

3.3. Would you use it? What makes an app an attractive option to support your journey as a carer of cancer a survivor? At what stage of the journey with the disease would an app be most useful to you and to your patient? (for example, during active treatment or after follow up).

**4. Focusing specifically on follow up, how can the app improve some aspects of the follow up?**

Prompts: ask about appointments, live tracking of the patient’s journey in the clinic, medication refill, waiting to get your medication ready? Medication shortage, how the app can help you to communicate with HCPs about such issues?

**5. With regards to emotional support, how can the application provide an emotional-social support to cancer patients?**

Prompts: ask about support groups, to what extent in Jordan people like to share their experiences via written and visual content (videos) or simply group meetings with peers? What are your thoughts on that? (prompts, why, explain, other opinions? etc)

What are your recommendations for the content and information needs?

What are the social and individual barriers that may facilitate/hinder using apps for social support?

**6. What about the disadvantages’ or pitfalls of a mobile app for cancer supportive care? Will you use it all the time or at certain times?**

prompts: can you explain why would you continue using it?

Prompts: what is the perceived value of different features? Do you think that all features will be useful during all phases of the disease? (Y/N)

Do you think that the mobile app can replace face to face consultation or enhance communications? (Y/N)

**7. Is there anything else you would like to share with us on the topic?**
