# Supplementary material for: Cancer literacy among Jordanian colorectal cancer survivors and informal carers: Qualitative explorations
Source: Front Public Health. 2023 Mar 20;11:1116882. doi: 10.3389/fpubh.2023.1116882 (PMC10067669; doi:10.3389/fpubh.2023.1116882)
Supplement: Supplementary file 3 [file Table_3.DOCX]

**Manuscript: Cancer literacy among Jordanian Colorectal Cancer Survivors and Informal Carers: Qualitative Explorations**

**Consolidated Criteria for Reporting Qualitative Research (COREQ) 32-item checklist:**

**Developed from:**

**Tong A, Sainsbury Craig J. Consolidated criteria for reporting qualitative research (COREQ): a 32-item checklist for interviews and focus groups. *International Journal for Quality in Health Care.*2007, Volume 19, Number 6: pp.349-357**

| **COREQ Criteria** | **Criteria fulfilment in the current research** |
| --- | --- |
| **Domain 1: Research team and reflexivity** | |
| **Personal Characteristics** | |
| 1. Interviewer/ Facilitator | It was noted in the methodology section that the first author (SJM) performed the focus groups and interviews. |
| 2. Credentials | In the methodology section, the research team's qualifications were listed. |
| 3. Occupation | The research team's occupations were given in the methods section. |
| 4. Gender | The methods section noted that the analysis team comprised of three female academics. |
| 5. Experience and training | The analysis was undertaken by a research team with rich experience performing qualitative research in healthcare settings, as stated in the methods section. |
| **Relationship with participants** | |
| 6. Relationship established | This was not stated in the study. Before the study, none of the research team had relationships with any subjects. |
| 7. Participant knowledge of the interviewer | SJM phoned all eligible participants to clarify the research objectives and answer any questions, and because the interviewer and CRC survivors did not know each other, additionally a brief explanation of the research was given during face-to-face interviews. Caregivers were informed of the study's objectives prior to the Skype focus groups. Researchers emailed or WhatsApped participants a research information sheet. This was in methodology section. |
| 8. Interviewer characteristics | Qualification, occupation, gender, and the absence of a pre-existing link between the interviewer and the interviewees are all provided about the interviewer. The methods section addressed this point. |
| **Domain 2: Study Design** | |
| **Theoretical Framework** | |
| 9. Methodological orientation and theory | Descriptive phenomenology was the main theoretical orientation. Data was analysed, however, using a thematic framework. This was noted in the methods section. |
| **Participant selection** | |
| 10. Sampling | A convenience sampling strategy of ambulatory CRC survivors and carers was used, with participants recruited from a large tertiary hospital (JUH) utilising an indirect recruitment strategy by their specialists, as detailed in the methods section. |
| 11. Method of approach | Clinicians approached participants in person or by phone. This was stated in methodology. |
| 12. Sample size | This study included 15 cancer survivors who were interviewed. As stated in the methodology section, sample size was chosen by data saturation and a stopping threshold of three interviews. Three focus groups (comprising ten carers) with carers were held, and talks were halted when no new fresh ideas for topics were created, indicating thematic saturation. |
| 13. non-participation | All eligible participants who were approached and agreed to participate in accordance with the procedures described in the methods section were enrolled. A flow chart outlining the recruitment process for CRC survivors was included in the methods.  Thus, the paper did not discuss non-participation. |
| **Setting** | |
| 14. Setting of data collection | Face-to-face interviews with CRC survivors were held in a hospital conference room, while focus groups with informal carers were held through Skype. This was mentioned in methodology. |
| 15. Presence of non-participants | Nobody other than the participants in the focus groups and interviews was present. |
| 16. Description of sample | Table 2 shows the sample characteristics of CRC survivors, while Table 3 shows the characteristics of focus group members. Mentioned in the results. |
| **Data Collection** | |
| 17. Interview guide | The interview guide were provided as a supplementary file(s) .(Please refer to supplementary material 2 for CRC survivors' interview topic guide and supplementary material 3 for focus groups topic guide). |
| 18. Repeat interviews | There were no follow-up interviews, as noted in the methods section. |
| 19. Audio/ visual recording | As stated in the methods, all face-to-face interviews with survivors and online focus groups conducted via Skype were audio-recorded. |
| 20. Field notes | During the individual interviews, only handwritten notes were taken. |
| 21. Duration | The length of the individual interviews and telephone focus groups was specified in the methodology section. |
| 22. Data saturation | Saturation of data was used to determine sample size, and this was documented in the methods section. |
| 23. Transcripts returned | For participants to review and remark on, transcripts were not given back. |
| **Domain 3: analysis and findings** | |
| **Data analysis** | |
| 24. Numbers of data coders | First author performed data coding. However, two authors separately inspected the original author's coding, and disagreements were discussed. All authors thoroughly debated and approved the coding framework. |
| 25. Description of the coding tree | The "Methods" section describes the code structure and framework. |
| 26. Derivation of themes | Themes were derived inductively/deductively from data and literature review. |
| 27. Software | The NVivo 12 software was used to manage and code the data. |
| 28. Participant checking | This was not carried out. |
| **Reporting** | |
| 29. Quotations presented | In the results section, participants' quotes were included, and a complete list of findings is in supplementary material 4. |
| 30. Data and findings consistent | To verify interpretations and findings, all authors evaluated and validated the final themes and subthemes. |
| 31. Clarity of major themes | The thematic framework weighted all themes equally. |
| 32. Clarity of minor themes | Within the thematic framework, all themes received equal weighting. |
