# Supplementary material for: Cancer literacy among Jordanian colorectal cancer survivors and informal carers: Qualitative explorations
Source: Front Public Health. 2023 Mar 20;11:1116882. doi: 10.3389/fpubh.2023.1116882 (PMC10067669; doi:10.3389/fpubh.2023.1116882)
Supplement: Supplementary file 4 [file Table_4.DOCX]

**Supplementary 4: CRC survivors' and informal carers' focus group excerpts**

| **Additional quotes for theme 1 “Current state of information provision and counselling”** | |
| --- | --- |
| ***2^nd^ order (empirical) codes*** | ***Example quotation*** |
| **Subtheme 1A: Format and quality of information** | |
| Underuse of patient directed materials | - *“Usually, they explain everything to the patient or his family verbally and provide us with the medical report that can be understood by people with medical background because it is for medical use, so overall, many patients don't have deep and detailed knowledge about their condition and I the patient believe they should have given us a personalised plan with the stage, treatments, labs, and everything in a language the patient understands. “(IC male,26 years)* - *“I don't know a lot about the disease since when my doctor told me they found a tumour, he didn't tell me about my stage. They sent me my medical report so that I could apply for insurance, and even though it is written in jargon and has all the details of my case, I couldn't comprehend a word of it. After the procedure, the doctor informed me that the bone scan from the CT scan was normal and that 58 benign nodes had been removed for the biopsy, leaving only one that was thought to be disease-affected.” (CRC survivor male,72 years)* |
| Information supplied verbally | - *“Some doctors give me accurate and detailed information about my health, especially when it comes to surgery. Other doctors, on the other hand, don't give size information or details. Instead, they just give headlines or basic information. I usually look at the pamphlets that come with the medicine to see if there are any side effects. “(CRC survivor male,72 years)* - “*All counselling was done orally.” (CRC survivor male,72 years)* - *“I asked my doctor if he wouldn't mind having the consultations recorded after a time because I used to forget when I got home, especially if I went to the doctor alone.” (CRC survivor male,56 years)* |
| Inconsistent supply of written information | - *“I only had the medications, and they had written instructions on how to take them. After reading the leaflets and becoming horrified by all the side effects, I asked the Community pharmacist about this medication. He said that it can lead to addiction, so I decided not to take it even though my doctor had prescribed for the numbness it because I am not used to and dislike taking medications.” (CRC survivor male,56 years)* - *“Handouts with information about what to eat after surgery were given to me, but my family was in charge of everything. But after the doctor fixed the ostomy, no plans were given.” (CRC survivor male,53 years)* - *“My doctor told me to eat a healthy diet, but he didn't tell me what to eat. I used to eat a balanced Mediterranean diet, so I don't think this is a big deal. However, I avoided some foods that upset my stomach so that my bowel habits wouldn't change. “(CRC survivor, female 59 years)* - *I only got advice about my diet from my doctor, who told me I could eat a normal diet. When I got out of the hospital, I didn't get any special nutritional counselling.” (CRC survivor male,56 years)* - *“Handouts with information about what to eat after surgery were given to me, but my family was in charge of everything. But after the doctor fixed the ostomy, no plans were given.” (CRC survivor male,53 years)* |
| Patient friendly content | - *“The contents should assist them continue treatment and follow up; there is hope. It must distract them from worrying about whether the cancer is spreading or returning, so, the massages should be positive and not make them think about their condition, because all they know about cancer is that it's deadly….. for example. The design and contents must send positive emotional and spiritual massages through the way the information is given and received by the patients. Some will accept watching a video about a surgery, while others will be bothered and disgusted. It will be better if such contents are viewed as a cartoon or animation video.” (IC male,32 years)* - *Doctors do their best to explain things in lay terms, but many patients still don't obtain all the information they need since there aren't enough patient-friendly resources available. Even though I'm a doctor, I started learning more about my stepmother's condition.” (IC female,41 years)* - *“Because not everyone is a doctor, it should be basic and easy to understand.” (IC male,26 years)* - *As soon as I left the hospital after my first surgery, my son brought me books and CDs about colorectal cancer. Teaching aids regarding cancer can be found here. In addition to providing general information, it teaches you how to better control your nutrition. But the amount of content is so extensive that I am unable to read everything on my own, also my condition is complicated stage 4 and the information doesn't suit my current needs” ... (CRC survivor, female,61 years)* |
| Flashcards, push notifications, videos, podcasts | - *“If there are videos or animations, I think most patients would like them, especially if they are about the disease.” (CRC survivor, female 53 years)* - *"I recommend that you add content to the app for the general public to increase awareness of CRC via push notifications, and that you make it a nationwide app so that people may receive SMS or text notifications inviting them to screening events."(IC female,41 years)* - “*Most people in our society don't like to read, so it's important to make sure they get timely information in an easy-to-understand way. This stops individuals from doing online research and coming back with conflicting information that might delay their treatment or affect their decision-making. My family and I decided with the doctor to tell my father about his situation gradually and cautiously over the course of two months following the surgery.” (IC female,27 years)* - *“I prefer watching videos because I don't like reading...” (CRC survivor male,72 years)* - *“I think even podcasts are a good idea because many people don't like to read.” (CRC survivor male,56 years)* - *“Because not everyone is a doctor, it should be basic and easy to understand.” (IC male,26 years)* |
| **In-depth readings** | - *“As for me, I prefer to read comprehensive, in-depth, evidence-based materials. The information should also be patient-focused and simple to grasp because many patients are unaware of their stage or the therapies they are receiving, which prevents them from expanding their knowledge.” (CRC survivor, male 58 years, university)* |
| **Subtheme 1B: Paternalistic model of information delivery and acquisition based on age and education** | |
| **Didn’t know what to ask during consultations** | - *“The doctors were wonderful and took the time to explain everything to me; my son signed the consent form because I was unable to do so; we were given a copy of the form; and all of my medical records are in my possession. “(CRC survivor male,72 years)* - *“…But I don't always know what to ask for, so I don't get the information I need when I need it. Other times, I forget after I go home.” (CRC survivor male,56 years)* |
| **Developing cancer literacy slower than the pace of care** | - *“The diagnosis came out of the blue, and I was taken aback; I didn't know what questions to ask, so I just listened while the physicians laid out my treatment options.” (CRC survivor male,56 years)* - *“During treatment, it was hard, and I couldn't have made it without the help of my doctors and family. At the time, my main goal was to get better, so I didn't think about the consequences. Now, I'm fully responsible for my health, and I feel like I need to take an active role in managing my life.” (CRC survivor male,56 years)* |
| **Other barriers for literacy**  **Building**  ***Fatalistic beliefs***  ***Language info access barriers*** | - *“No one knows what causes bowel cancer, and I don't believe that the food we eat has anything to do with it.” (CRC survivor, male 65 years)* - *“Perhaps adhering to particular diets might help prevent cancer, but now that I have the condition, I believe that diet has a limited impact right now.” (CRC survivor, female 54 years)* - *“I had rectal cancer and had to go to the restroom a lot. It was distressing, so I stayed home by the toilet. This happened after surgery, and my doctor stated it was normal, but I couldn't deal with it until he told me I had anterior resection syndrome. I looked up these words online and contacted a relative for English help. I translated several words to Arabic...” (CRC survivor, male 67 years)* |
| **Varied information experiences of carers** | - *As a carer and ophthalmologist, I’ve had to continually brush up on my knowledge and learn new things; for instance, I can't remember how to apply a surgical dressing since I haven't done it in a long time. Also, as you know, there are time limits, limited educational resources, and even people with higher education may not know much about cancer.”* *(IC female,41 years)* |
| **Doctor-patient communications** | - *My doctor told me it's stage 2, but I didn't ask him what that means or if its's going to make a difference for me to know. “(CRC survivor male,56 years)* - *“I honestly don't know [cancer stage], since they do investigations and tell me they are doing this and that. I didn't choose which therapy to do. I just go to my doctor, and he tells me what to do.  After four cycles, I'm not sure why the doctor recommended a colonoscopy. “(CRC survivor male,72 years)*   *“But it's pretty surprising that after what appears to be chemotherapy [FOLFOX] I haven't lost any hair, and I'm not sure why”. (CRC survivor male,56 years)*   - *“When I was eventually sent to the correct doctor (the surgeon), I froze up and didn't ask any more questions; I was exhausted and overwhelmed by the ordeal that all I wanted to do was wake up from this nightmare.” (CRC survivor male,72 years)* - “*I felt like time was running out, so I told the doctor to do whatever he needed to do to save my life. After four years, I'm rethinking my choices. Maybe I didn't or couldn't think properly at the time. I feel like my whole body has changed because I have a permanent colostomy, but in reality, I had no choice but to go along with what the doctor had planned for me.” (CRC survivor, male,56 years)* - *“I have complete faith in my surgeon, and if he tells me to jump out of a window, I will.” (CRC survivor, female,61 years)* - *Another stated “The doctor's reputation, along with his ethics and experience, is the most important thing. Half of the success of the treatment is down to the doctor.” (CRC survivor male,72 years)* |
| **Ill-timed information acquisition** | - “*My doctor told me it's stage 2, but I didn't ask him what that means or if its's going to make a difference for me to know.”* *(CRC survivor male,56 years)* - *“For example, when I notice a new symptom, I notify my doctor, who advises me on what to do and why. At home, I regularly forget or miss things. I made the decision to jot down all of the key points and notes from my doctor visits. My physicians' notes fill a notepad. The doctor seldom asks me if it was resolved until my follow-up appointment, and occasionally I forget to mention it.” (CRC survivor, male 58 years)* - *“Yes, they gave me booklets about colorectal cancer at the start, which were helpful at the time. But as my treatment went on and it's been three years, my needs have changed, and the hospital only cares about clinical follow-up. The rest is up to the patient's awareness, preferences, and personal efforts. “(CRC survivor, male 58 years)* |
| **Ageist communication barriers** | - *“During early consultations my son and daughter were asking the doctor about the treatment plan and after the surgery when I got home, they didn’t tell me its cancer, just only before chemotherapy they told me everything.” (CRC survivor male,72 years)* - *“My dad didn't find out what was wrong with him until three months after his surgery. Together with his doctor, we decided to tell him slowly about his case to keep his spirits up and avoid any emotional trauma. As we go along, he learns more. But I think he needs reassurance and support right now...”  (IC female,27 years)* - *“My mum is 78 years old; she was diagnosed with breast cancer for the first time 10 years ago, but we kept the news from her until now by telling her that the tumour was actually a "benign gland." After the second round of chemotherapy, however, she began to think something was wrong and collapsed in a state of fear, so I urged her doctor to keep our conversations about her illness private and that I would inform her myself. Elderly people won't understand, when they become older, they catch life with both hands. She got colon cancer again, but simply had surgery…” (IC female,57 years)* - *“My mom has hearing problems and had a stroke, so we and her physicians decided not to tell her that she had cancer. As she depends on us (her children) for follow-up care and communication with the doctors, we have been hesitant to inform her up to this point.” (IC male,32 years)* |
| **Online resources**  **(Internet & social media)** | - *“I think it's better for the patient to get information from more reliable sources, like the websites of cancer centres.” (CRC survivor, male 58 years)* - *“It's impossible for me to continuously seeing my doctor, so I turn to the internet if there are any symptoms to hunt up information about. However, most of the time I just discover generic advice rather than the specific information I need.” (CRC survivor male,56 years)* - *Now it's up to me to take care of myself, which is why I started learning about my symptoms I didn't find many helpful things on the internet, like diet plans for people with colorectal cancer based on their stage and procedure. “(CRC survivor male,56 years)* - *“I checked groups on Facebook for colorectal CA support but I only found English speaking forums so I couldn't Join them because of language barrier.” (CRC survivor male,56 years)* - *“Even if they are Arabic speakers, I would also be cautious because I don't know them, maybe if it is a moderated forum, I will think about it.!” (CRC survivor male,56 years)* - *“It depends on the patient, and each patient has different skills. I also don't know where to find additional information.” (CRC survivor male,56 years)* - *“I saw a nutritionist on you tube, she is very famous, and she recommended that I begin an intermittent fasting diet similar to the Keto diet, since she claimed that fasting will help to eliminate cancer cells. No white flour, sugar, bread, pasta, potatoes, rice, or maize. However, when I learned that the ketogenic diet is based on a high-fat diet, I informed the oncologist. He became upset and said that this is definitely not recommended for you.” (CRC survivor female,58 years)* - *“I tried to find information on the Internet, but I haven't found what I need. I searched for "long-term side effects of colon cancer chemotherapy," but there were so many results that I got confused and stopped looking. I don't know how to search for things that support my case.” (CRC survivor male,56 years)* - *I didn't find many helpful things on the internet, like diet plans for people with colorectal cancer based on their stage and procedure. “(CRC survivor male, 56 years)* |
| Social circles  (Family, friends and colleagues) | - *“When I tried to translate my medical records to Arabic using Google Translate, I couldn't comprehend what I was reading, so I approached a family member who is a doctor to help me. If you don't work hard to comprehend, you won't acquire a comprehensive and thorough grasp of your diagnosis, so the ball is in your court. “(CRC survivor, male 67 years)* - *“Doctors need to explain all the results more clearly, especially when providing us with medical records. I don't always understand what's written down. However, my son is a nurse, so I frequently ask him to clarify things to me.” (CRC survivor, male 65 years)* - *“When it comes to learning more about the condition in general, my granddaughter turns to Google. The younger generation may be technologically more adept than we are.” (CRC survivor male,72 years)* - *“Some doctors give me accurate and detailed information about my health, especially when it comes to surgery. Other doctors, on the other hand, don't give size information or details. Instead, they just give headlines or basic information. I usually look at the pamphlets that come with the medicine to see if there are any side effects. I also ask my daughter, who is a nurse, to help me and explain anything I don't understand...” (CRC survivor male,72 years, high school)* - *“I didn’t know about any support groups and my doctor didn't tell me about them. I also couldn't find any online support groups.” (CRC survivor male,56 years)* - *“The doctor was understanding and supportive during my treatment, but afterward, I felt like I had to adjust to my new life. I needed my friend's [CRC survivor] help, and he told me what he did next. For instance, he told me that after a year, you would either have an MRI or they would recommend one because we both received the same treatment. Such peer support is a blessing. “(CRC survivor male,56 years)* |
| **Subtheme 1D: Need for awareness** | |
| **Reflections of behaviours during treatment** |  |
| - *Need for active participation* - *Regretting putting off seeing care provider* | - *“My doctor said I needed a colostomy that would be permanent. Although it was difficult, I had to accept it since I believe in God and know that I cannot undo the past. It was then that I made the conscious decision to educate myself about colostomy care and adapt my lifestyle accordingly. Now, my colostomy is just another part of me.” (CRC survivor, male,56 years)* - “*While I was going through the treatment, all I wanted to do was finish it... If I knew someone who had been there before me, I would have asked them, but I couldn't find anyone who had been there before me. “(CRC survivor male,56 years)* - *“I regret making the poor decisions that led to the delay in diagnosis, and by the time I found the appropriate healthcare professional (the surgeon), I was exhausted and overwhelmed and simply wanted to put this nightmare behind me.” (CRC survivor male,72 years)* - *“I didn't go to the hospital right away because I was worried about my kids and didn't know where to put them because I was so busy. I also didn't pay attention to my symptoms until they got really bad and scared me.” (CRC survivor male,36 years)* - *“The worst thing was, in particular, the way to identify the problem. If only I had recognised the symptoms earlier or hadn't ignored them, I believe I would have found the correct doctor at the right time”. (CRC survivor, male 59 years, university)* - “*Even when I was pregnant and after I had my children, my iron and haemoglobin levels were low. I'm used to having low iron levels, but I didn't know if haemoglobin levels of 6 mg/dl are normal or not! At a public hospital, I was given tests and blood. They told me to stay in the hospital so that they could figure out why my blood levels were so low. After signing the consent form, I left the hospital completely on my own. I ran away from the hospital in February because I didn't want to stay there and I needed to take care of my kids.” (CRC survivor male,36 years)* - *“I was oblivious of how bad the illness was also because I used to come here because I follow up with a gynaecologist for IVF, so I am familiar with the staff and feel more at ease in this environment. I didn't even ask why my blood level is six or what it means. This could be because I don't fully understand what it means.” (CRC survivor female,36 years).* |
| CRC reframed as a chronic illness   - CRC national screening program - Cancer family communications and genetic counselling | - *“If I had known that I had symptoms such as chronic constipation, which is linked to colorectal cancer, But I believe that I was mistaken because I did not see a doctor to investigate the cause of my constant constipation”. (CRC survivor male,72 years)* - *“Cancer is everywhere, Air and water Pollution, plastics, chemicals in food and everyday home products. Awful. Too bad most doctors don't know and cannot advise people on actual specific prevention”, (CRC survivor male,57 years)* - *“I suffered from persistent constipation for three to four years. I tried everything to get rid of it, but nothing worked. In the UAE, I was constipated, and laxatives did not help. I had several friends from other countries who gave me natural cures to heal me. I frequently go three weeks without being able to defecate in the restroom. Also, I had no idea how dangerous it was to be constipated for an extended period of time until it occurred to me. I had no idea it may progress to something more serious, such as cancer. After a time, I found myself unable to focus on many of the things going on around me. I was advised to see a doctor since I had accumulated toxins in my body... “(CRC survivor, female,61 years)* - *“Cancer awareness activities should be included in educational programmes and more information should be provided to patients and the general public about health concerns, including cancer. “(CRC survivor male,72 years)* - *“I learnt about breast cancer screening via TV ads and a social media campaign, but I never considered that there is a similar test for CRC." (CRC survivor, female 59 years)* - *“Many people have cancer symptoms but don't realise it since they don't know enough about it.” (IC male,35 years)* - *"After discussing family history and the risk of first-degree relatives developing the illness, my medical oncologist requested me to bring my family to the next meeting to discuss surveillance. Since I received my diagnosis at age 36, I told my parents and siblings and did everything I could to spare them the same trauma. It was challenging to inform my loved ones they had a higher cancer risk and should be screened since a voice in my head told me I was imparting bad news. " (CRC survivor, female 59 years)* |
| - Coping, support & adjustment to cancer-free stage | - *“Based on what I’ve been through, I think we need a more organised way to give patient counselling. I didn't know anything about the disease, and all I knew about chemotherapy was that it caused hair loss…..I didn't know how to live after cancer, so I missed some details.” (CRC survivor male,56 years)* - *“I now lead a very routine life and walk for an hour each day. Adding extra fruits and vegetables to my diet was another modification I made. YouTube and local TV programmes like Seven Doctors are where I acquired my diet guidance….. I used to love eating meat, but I’ve cut back recently. Because I am aware that people may consume less protein as they age. “(CRC survivor male,72 years)* - *“My wife takes care of me in every manner and was quite supportive during my treatment and difficult times. She's uplifting. So, my daughter (a nurse) explained my medicines and medical reports if I had questions.” (CRC survivor male,72 years).* - *“Until my father got cancer, we had never seen anything like this in our family history, thanks be to God. Dad was doing great when he was diagnosed, and now he's our first cancer patient." When you hear the term "cancer" for the first time, you realise what a terrifying experience it is. It's excruciating…. “(IC male,26 years)* |

| **Additional quotes for theme 2:” Impact of lack of information, awareness and literacy.”** | |
| --- | --- |
| ***2^nd^ order (empirical) codes*** | ***Example quotation*** |
| **Subtheme 2A: Symptoms appraisal and medical help seeking** | |
| Maintaining normalcy | - *“Peppermint, sage, local herbalists, and recipes from the downtown herbalists were some of the things I attempted to alleviate my bloating*.” *(CRC survivor, female 54 years, High school)* - *“Because of stomach discomfort, I thought I had “H pylori” for seven months. I attempted to look up the symptoms on Google, but it wasn't very helpful. I took pills from the local drugstore.” (CRC survivor, male 59 years)* - *“The story began two years ago (23/6/2018) with abdominal pain that was not severe but persistent and chronic, so I assumed it was a cold or normal and would go away, but after two months the pain intensified, so I went to the primary care, and they gave me medications because they believe it is abdominal cramps when I drink yoghurt. I used to be always tired and have stomach problems. “(CRC survivor, female 53 years,* - *“I had been taking Imodium from the local drugstore to manage my loose stools at work, assuming it was IBS, and I didn't seek medical assistance until I started vomiting up... I didn't know about it.” (CRC survivor male,56 years, university)* - *“I used to go to an herbalist to receive folk remedies to cure my constipation, which worked for a while, but several of my friends advised that I try something else…”* *(CRC survivor, female,61 years)* - “*I used many types of laxatives and natural remedies, for 3 years to get over my long-standing constipation.”* *(CRC survivor male,72 years)* |
| Interpreting as “alarming symptoms” | - *Before I had to visit the restroom to poop every nine days owing to chronic constipation.” (CRC survivor male,72 years)* - *“We wouldn't know my dad has cancer if I wasn't a nurse. Anaemia is connected to colon cancer, which many people don't realise. These symptoms are vague. My father doesn't know the signs of this condition or what to do if he experiences any of them, so when they first appeared, none of us knew that a colonoscopy was necessary. We'd be late without this knowledge. Late diagnosis means difficult treatment and poor outcomes.” (IC male,35 years)* - “*I thought it [Bloody diarrhoea] was from food so ignored it but then it happened every time I went to the bathroom, so I realised it couldn't be normal.” (CRC survivor male,56 years, university)* - *“I was trying to lose weight and thought I was getting healthier when, out of the blue, I was told I had cancer” (CRC survivor female,33 years)* |
| Routes to diagnosis | - ***Self-led.*** *“When I went to a new doctor, she offered me medication for the pain, but it didn't work. Another private doctor I visited recommended a colonoscopy because I'd never had one. I followed what he told me and had one at a private hospital. After seeing the alarming results, the private clinic doctor advised Prof. X.” (CRC survivor, male 59 years)* - ***Multiple points of care*** “*The primary care clinic where they examined me, diagnosed me with irritable bowel syndrome, and gave me a pain reliever. When I drink water, it has no effect on me, but when I eat something, my stomach begins to ache all the time.” (CRC survivor, female 53 years)* - ***Multiple points of care”*** *I attended my city's hospital and had a right-side x-ray done. After gallstone surgery, I still felt stomach discomfort. I went for a follow-up visit for suture removal surgery and told the doctor I don't believe the gallstones were removed because I still have right side discomfort and haven't improved since the week before my colonoscopy at this hospital. Blood in my faeces led me to get a colonoscopy. I didn't expect to get cancer. I urged the doctor to tell me everything and explain everything since I am educated and want to know everything about my condition. The doctor arrived and said, "During the colonoscopy, we discovered that you had a tumour and an abscess." (CRC survivor****,*** *male 61 years)* - ***Multiple points of care*** *“The discomfort got worse, though. A week later, I couldn't walk because I felt like I was in labour. My spouse questioned why I was in bed, and I said I was in excruciating pain. The pain was tolerable for seven months before intensifying.” (CRC survivor, female 53 years)* - ***Multiple points of care*** *“But in August, I experienced severe stomach pain. I once liked labour pain, but it was horrible. I called my cardiologist doctor because I thought I was taking a cardiac medicine that caused red blood cells to break regularly…… “(CRC survivor, female 59 years)* - ***Emergency admission*** *"I* *was on a strict diet for one year because I weighed 91 kg. I lost weight and got down to 78 kg. When I went back to eating the way I usually do, my stomach hurt a lot. I couldn't even stand up or take a sip of water, and I felt like I was going to throw up. My pain was so bad that we came to the emergency room, where they did tests that led to the diagnosis of cancer”. (CRC survivor female,33 years,)* - ***Emergency admission****: Before April 2017, I had bloody stools. Fresh, painless blood. I thought it would be a little issue, but it lasted two weeks. Blood faeces was painless. Because of this, I googled the symptoms and hurried to JUH's ER. The doctor told me I had rectal cancer.” (CRC survivor male,56 years)* - ***Emergency admission****: “I couldn't even walk because I was so exhausted, and I kept falling down. My daughter advised me to have a check-up at the hospital since she did not know what was wrong with me. I went to the hospital and had a blood test, which showed my haemoglobin level was 6. They began investigating…” (CRC survivor, female 59 years, secondary)* |
| **Subtheme 2B: Treatment experience and managing side effects** | |
| Unmet needs | - “*Someone helped me practise ostomy care after the doctor taught me. He trained my son to change and care for his colostomy bag”. (CRC survivor male,53 years, university)* - *“Because I worry about the bag leaking at work and my colostomy base not always being properly in place, I’ve opted to seek expert help once or twice, despite the cost. I was afraid it might fill up too fast and explode. It may cause social embarrassment if I am unaware of the odour or a sudden explosion. It was initially susceptible to being knocked out of place by even the slightest movement, but I eventually learned how to deal with it...” (CRC survivor, male,56 years)* - *“I didn't have to leave the house very often while I was recovering from surgery in the summer since I was going to the restroom so often. I had to take some time off without pay since I couldn't go back to work until I felt better.” (CRC survivor male,56 years, university)* - *“I also have trouble bending over and putting on socks till today... For instance, I can't wash my legs. Now it's a lot better, but I still can't do it like I used to. After surgery, I have made only around 60-70 percent of the improvement that was expected, and I still have trouble performing even the most basic of duties. (CRC survivor, male,56 years)* - *“I couldn't go back to the hospital for stoma help and had no one to call, so I phoned a cousin who knew a nurse. He came to provide extra hands-on training.” (CRC survivor, female 54 years)* - *“Because I worry about the bag leaking at work and my colostomy base not always being properly in place, I’ve opted to seek expert help once or twice, despite the cost. I was afraid it might fill up too fast and explode. It may cause social embarrassment if I am unaware of the odour or a sudden explosion. It was initially susceptible to being knocked out of place by even the slightest movement, but I eventually learned how to deal with it...” (CRC survivor, male,56 years)* - *“I didn't know where to find high-quality bags and bases at first.” (CRC survivor, male,56 years)* |
| Engagement with tertiary care | - *“ER visits are required when the patient isn't able to communicate his or her symptoms to the doctor or when we can't get in touch with them in time, because other facilities don't take cancer patients like us and we can't afford the costs of private treatment.” (CRC survivor, male 67 years)* - *“I didn't know who to call when I had side effects from chemotherapy because I got it at a different place not this hospital. So, my family had to pick me up again to the ER.” (CRC survivor, male 58 years)* |
| Managing chemotherapy side effects | - *I took some kind of chemotherapy by mouth after surgery, but I can't remember what it was called... When I had been through chemotherapy, so I had no immunity, and my potassium levels were very low. I was alone because I couldn't move because of chemotherapy and radiation therapy. I fell when I tried to walk, and I had to be carried back to the hospital. So, I had to stay in the isolation room for a week or ten days before I could leave.” (CRC survivor, male,56 years)* - *“During chemotherapy, no one told me what I should or should not eat or drink; they simply gave me the regimen and sent me home. And I didn't press the matter further with them.” (CRC survivor male,72 years)* - *“They gave me chemotherapy, and they said it won't cause any hair loss, this is what I was concerned about, but now I have numbness in my feet that is getting worse…. “(CRC survivor male,56 year)* - *“We will have to deal with the bad effects because, in my opinion, they are caused by the therapy and can't be avoided. I have no power over them. I had terrible diarrhoea, so I had to go to the bathroom every 15 to 30 minutes. He told me I should eat soup and drink lots of water. He told me I should eat cooked vegetables instead of things that are high in fat. He also told me to drink a lot of water, especially while I was getting chemo.” (CRC survivor, male 58 years)* - *“During my therapy, I started to encounter adverse effects that my doctor had not previously informed me about. “(CRC survivor, female,61 years)* - *"We gave him veggies and fruits because we didn't know what diet to give him, but the doctor told us to give him rice and potato for the colostomy or to minimise the diarrhoea. If we understood colostomy care and what diet to provide a colon cancer patient, we also needed to know remedies and preventative actions.” (IC female,27 years)* |
| Distal side effects | - *I'm constantly on the verge of passing gas and my stools are becoming increasingly watery.  It's quite upsetting. “(CRC survivor male,56 years)* - *“The doctor said my burning feet were likely a side effect of treatment. I didn't know this and don't know how to stop it. My doctor didn't tell me that the numbness in my feet would be chronic*. *“(CRC survivor male,56 years)* - “*I'm pretty close to normal, but my stools are still soft. I have a mix of hard, soft, and loose bowels. If I eat something oily or fatty, my stools change right away. This can be annoying because I have to be close to the toilet. It took me a while to figure out what was going on. When I asked the doctor, he said it was normal, but he didn't tell me much about how to change my diet to deal with it.” (CRC survivor male,56 years)* |
| Previous cancer experience | - *“Since my daughter was diagnosed with facial tumour 14 years ago, my take on cancer is a there a chance that it will be treated like other diseases, which makes me want to fight it hard. I was able to learn a lot from what my daughter went through, I started learning about her condition to better support her.” (CRC survivor, male 58 years)* - *“Until my father (71 year) got cancer, we had never seen anything like this in our family history, thanks be to God. Dad was doing great when he was diagnosed, and now he's our first cancer patient." When you hear the term "cancer" for the first time, you realise what a terrifying experience it is. It's excruciating….” (IC male,26 years)* |
| **Subtheme 2C: Psychological impact, notably stoma-related** | |
|  | - *“My social and mental health worsened after my colostomy. Even though I knew it was temporary, I needed aid from my family with caregiving. Before, I was a strong, independent parent.” (CRC survivor male,53 years).* - *“It was a foreign insertion to my body, I couldn’t cope with it honestly and it got frequently inflamed, the doctor showed me how to take care of it but the problem was psychological…. Thanks God it was temporary...” (CRC survivor, male)* - *To put it simply, I was embarrassed by the colostomy, but the doctor assured me that it was just temporary and that he would be able to close it within 3 months, so I was patient and tried to deal with it. However, I wasn't very comfortable with it and the doctor advised me to maintain it for 9 months for better results.” (CRC survivor, male,53 years)* |

| **Additional quotes for theme 3:” Healthcare structure and its influence on literacy.** ” | |
| --- | --- |
| ***2^nd^ order (empirical) codes*** | ***Example quotation*** |
| **Subtheme 3A: Fragmented landscape of delivery** | |
| Multiple points of care | - *“I went to 3 hospitals to get my treatments. Each hospital has its own system and routines. Maybe the hospitals talk to each other, but it was hard for me to get treatments in different settings and with different teams. Sometimes I don't even know which care provider is taking care of me…..” (CRC survivor, male,56 years)* |
| Communication barriers | - *“The doctors at the other institution where I underwent chemotherapy did not check on me over the course of the treatment, and I was unable to meet the consultant because I only saw him at the start and end of the chemotherapy, so I made the decision to speak with the surgeon, who was more accommodating.” (CRC survivor, male,56 years)* - ***“****ER visits are required when the patient isn't able to communicate his or her symptoms to the doctor or when we can't get in touch with them in time, because other facilities don't take cancer patients like us and we can't afford the costs of private treatment.” (CRC survivor, male 67 years)* - *“…and I was unable to navigate where should I go and whom should I contact for financial arrangements and also sometimes contacting providers, collecting information from here and there was challenging, without my wife, my cousins and some friends I couldn't make it. “(CRC survivor, male,56 years)* |
| **Subtheme 3B: Governmental bureaucracy and financial arrangements** | |
| Need for information | - *“When they [the ministry of health] know you have cancer and require treatment, they will give you a priority for therapy coverage and free treatment, there are no predetermined plans or definite treatment pathway for the patient. As a result of this, I had to deal with a lot of people to be transferred from military services to cancer centre. “(CRC survivor male,53 years)* |
| Holistic patient journey planning | - *The ministry of health pays for cancer treatment, which is a good thing, but the patient can't choose where to get care because it depends on formal arrangements. (CRC survivor male,53 years)*      - *My journey was full of uncertainties not only the disease findings and how waiting times were difficult and made me worried, but also the treatment pathway is complex.” (CRC survivor, male 59 years)* |
